# Toward a Compassionate Intersectional Neuroscience: Increasing Diversity and Equity in Contemplative Neuroscience

**DOI:** 10.3389/fpsyg.2020.573134

**Published:** 2020-11-19

**Authors:** Helen Y. Weng, Mushim P. Ikeda, Jarrod A. Lewis-Peacock, Maria T. Chao, Duana Fullwiley, Vierka Goldman, Sasha Skinner, Larissa G. Duncan, Adam Gazzaley, Frederick M. Hecht

**Affiliations:** ^1^Osher Center for Integrative Medicine, University of California, San Francisco, San Francisco, CA, United States; ^2^Neuroscape Center, University of California, San Francisco, San Francisco, CA, United States; ^3^Department of Psychiatry and Behavioral Sciences, University of California, San Francisco, San Francisco, CA, United States; ^4^East Bay Meditation Center, Oakland, CA, United States; ^5^Department of Psychology, University of Texas at Austin, Austin, TX, United States; ^6^Division of General Internal Medicine, University of California, San Francisco, San Francisco, CA, United States; ^7^Department of Anthropology, Stanford University, Palo Alto, CA, United States; ^8^School of Human Ecology and Center for Healthy Minds, University of Wisconsin–Madison, Madison, WI, United States

**Keywords:** meditation, interoception, neuroscience, diversity, community engagement, intersectionality, mindfulness, machine learning

## Abstract

Mindfulness and compassion meditation are thought to cultivate prosocial behavior. However, the lack of diverse representation within both scientific and participant populations in contemplative neuroscience may limit generalizability and translation of prior findings. To address these issues, we propose a research framework called *Intersectional Neuroscience* which adapts research procedures to be more inclusive of under-represented groups. Intersectional Neuroscience builds inclusive processes into research design using two main approaches: 1) community engagement with diverse participants, and 2) individualized multivariate neuroscience methods to accommodate neural diversity. We tested the feasibility of this framework in partnership with a diverse U.S. meditation center (East Bay Meditation Center, Oakland, CA). Using focus group and community feedback, we adapted functional magnetic resonance imaging (fMRI) screening and recruitment procedures to be inclusive of participants from various under-represented groups, including racial and ethnic minorities, gender and sexual minorities, people with disabilities, neuropsychiatric disorders, and/or lower income. Using person-centered screening and study materials, we recruited and scanned 15 diverse meditators (80% racial/ethnic minorities, 53% gender and sexual minorities). The participants completed the EMBODY task – which applies individualized machine learning algorithms to fMRI data – to identify mental states during breath-focused meditation, a basic skill that stabilizes attention to support interoception and compassion. All 15 meditators’ unique brain patterns were recognized by machine learning algorithms significantly above chance levels. These individualized brain patterns were used to decode the internal focus of attention throughout a 10-min breath-focused meditation period, specific to each meditator. These data were used to compile individual-level attention profiles during meditation, such as the percentage time attending to the breath, mind wandering, or engaging in self-referential processing. This study provides feasibility of employing an intersectional neuroscience approach to include diverse participants and develop individualized neural metrics of meditation practice. Through inclusion of more under-represented groups while developing reciprocal partnerships, intersectional neuroscience turns the research process into an embodied form of social action.

## Introduction

Contemplative practices such as mindfulness and compassion meditation hold promise for building more cooperative and multicultural societies, as they may increase prosocial behavior (Leiberg et al., 2011; Condon et al., 2013; Weng et al., 2015; Ashar et al., 2016) and neuroplasticity of networks involved in emotion regulation and empathic concern (Mascaro et al., 2013; Weng et al., 2013; Klimecki et al., 2014). Although contemplative research values cross-cultural dialogues between meditation practitioners, scientists, and other stakeholders to produce the strongest work (Varela et al., 1991), cultural forces within academia may prevent inclusion of people who belong to minority groups, and scientific findings may therefore not represent the wider population (Sue, 1999; Henrich et al., 2010; Hamrick, 2019). Further, physical and mental health inequities exist in the U.S. depending on social identities such as race, gender, sexual orientation, and disability status outcomes (CDC, 2013), and contemplative interventions have the potential to reduce stress associated with these disparities (Woods-Giscombé and Black, 2010) and build multicultural communication skills and communities (Magee, 2019; Yang, 2017). However, within contemplative research, racial and ethnic minorities are under-represented in randomized control trials of mindfulness-based interventions (Waldron et al., 2018) and neuroscience studies of meditation using functional magnetic resonance imaging (fMRI; see Supplementary Information [SI] p. 2 and Supplementary Figure S1). In addition, racial and ethnic minorities may also belong to other under-represented groups such as sexual and gender minorities and people with disabilities.

Here, we propose an *Intersectional Neuroscience* framework within the context of contemplative neuroscience to address these issues. In this framework, an intersectional lens (Crenshaw, 1991; Cole, 2009; Cho et al., 2013) is applied to neuroscience research procedures and methods, which aims to make the research as diverse and inclusive as possible, particularly for people who belong to multiple marginalized identities (such as women of color). To develop and pilot the feasibility of this framework, we used (1) community engagement with a diverse contemplative community to increase cultural sensitivity of research procedures to various under-represented groups (Wallerstein and Duran, 2010), and (2) individualized multivariate fMRI methods that accommodate neural diversity and produce subject-specific brain maps and statistics (Norman et al., 2006) representing mental states during breath-focused meditation. By adopting and integrating these methods, we aim to embody a more compassionate research framework that includes diverse identities and creates more equitable relationships.

The Intersectional Neuroscience approach is informed by intersectionality, an analytic framework which recognizes that lived experiences include multiple aspects of identity (e.g., race, gender, sexual orientation, disability status), which may simultaneously experience marginalization from multiple systems of power that favor or privilege certain identities over others (Crenshaw, 1991; Cho et al., 2013). Intersectionality frameworks emerged from Critical Race and Feminist studies to understand, for example, the marginalized experiences of Black women which could not be fully characterized through the single framework of race or gender alone (Crenshaw, 1991; Cho et al., 2013). Psychologists are also incorporating intersectional frameworks, recommending inquiries that take intersectional identities and perspectives into account, thereby improving theoretical and empirical methods to understanding social identities and interactions (Cole, 2009).

Community-based participatory research (CBPR) is an ideal methodology with which to implement intersectional principles, as a “transformative research paradigm that bridges the gap between science and practice through community engagement and social action to increase health equity” (Wallerstein and Duran, 2010). CBPR is an overall research approach, which aims to equalize power relationships between academic and community research partners, given historical inequities between scientists and marginalized groups (such as in the Tuskegee experiment; Green et al., 1997). Core principles include genuine partnership and co-learning, applying findings to benefit all partners, and long-term partnership commitments (Israel et al., 1998), which are developed through accountability, cultural humility, and the capacity of academics to reflect on their personal and institutional power (Wallerstein and Duran, 2010). To equalize power differentials, community and academic perspectives are engaged throughout the research process to address cross-cultural understandings of external validity, evidence, language, business as usual within academia, sustainability of interventions, and lack of trust (Wallerstein and Duran, 2010). Similar frameworks are advocated within contemplative neuroscience between scientists and contemplatives (Lutz and Thompson, 2003). Importantly, the research process then becomes bidirectional with continuous communication and sharing of resources and credit, which replaces the *status quo* of one-way transmission of knowledge from researchers to participants (Wallerstein and Duran, 2010). In this way, the research process becomes a form of *embodied social action*, or a way to embed compassionate behavior into research. These processes result in more effective and sustainable solutions for communities who are empowered to be active partners in the research process and cultivating their health (Wallerstein and Duran, 2010).

To integrate intersectional approaches with neuroscience, we highlight using individualized neuroscientific methods that accommodate neural diversity and can therefore be inclusive of more people. A major barrier to inclusion for many fMRI studies are due to individuals having “non-normal” brain structure and function including left-handers, those with mental health conditions and neurological disorders, and older adults. This is due to the common analytic approach of mathematically normalizing individual brains by warping them to a group template, and then performing standard univariate analyses to find regions that show greater average activity across the whole group (Dumit, 2004; Huettel et al., 2014). This approach assumes that brain structure and function are similar across people, and the resulting data are brain maps that are thought to generalize across the population. Within psychology and neuroscience, idiographic approaches that highlight within-subjects designs are gaining traction in addition to nomothetic approaches (Barlow and Nock, 2009; Poldrack et al., 2015; Smith and Little, 2018). Although group averages are useful, there are important limitations regarding efficiency and flexibility of these designs, such as generality of results (Barlow and Nock, 2009). Further, small-N designs treat the individual as the replication unit by collecting more data within each participant, and can help inform measurement, theories and models, and lack of effective experimental control over error variance (Smith and Little, 2018). Within neuroscience, idiographic personalized neuroscience approaches are gaining traction, using methods such as functional connectivity (Poldrack et al., 2015), multi-voxel pattern analysis (Norman et al., 2006; Haxby et al., 2014), and representational similarity analysis (Kriegeskorte et al., 2008). For instance, in the MyConnectome project, dynamic characteristics of brain connectivity, gene expression, and metabolites are collected and analyzed within one individual over multiple time points (Poldrack et al., 2015).

Multi-voxel pattern analysis (MVPA) is an ideal method to use within an Intersectional Neuroscience framework. MVPA uses machine learning algorithms to (i) identify unique brain patterns for each mental state within each individual, and then (ii) use these brain patterns to estimate the presence of mental states in a separate task (Norman et al., 2006; Haxby et al., 2014), such as whether short or long-term memory is being represented during a working memory task (Lewis-Peacock and Postle, 2008), or empathic care or distress is activated in response to suffering (Ashar et al., 2017). The resulting data are then *estimates of the presence of mental states*, which are information *derived from* but not *reduced to* brain maps alone (Kriegeskorte et al., 2006). This moves the field forward in what information may be gleaned from neural data. By identifying brain maps associated with mental states (and not stopping there as the main dependent variable of interest), information from these brain patterns can be learned using machine learning to make inferences about which mental states are present and how they are functioning (Norman et al., 2006; Haxby et al., 2014). Importantly, MVPA makes no assumptions about brain normativity and can be conducted in native space. By harnessing neural variability as a *strength* to find within-subject patterns, more groups with “non-normal” brains can be included.

Individualized approaches such as MVPA may be well-suited to measure mental states during meditation, which are internal and fluctuating between various states (Hasenkamp et al., 2012; Weng et al., 2017). For example, in breath-focused meditation (a core meditation skill that cultivates stability of attention which supports interoception and compassion), attention is focused on sensations of the breath, until distracted by other internal or external stimuli, and then attention is returned nonjudgmentally to the breath. This practice is simple but not easy. Even in this simple practice, the focus of attention will fluctuate over time between attention to the breath, mind wandering, and engaging in self-referential processing (Hasenkamp et al., 2012; Lutz et al., 2015). In the EMBODY task (**E**valuating **M**ultivariate Maps of **BODY** Awareness), we trained MVPA classifiers with fMRI data to recognize five foci of internal attention states (breath, feet, mind wandering, self-referential processing, sounds), which were directed via audio instructions (Weng et al., 2020). Unique brain patterns for each condition were recognized in 14 out of 16 participants (87.5%, including 8 experienced meditators and 6 novice controls). These brain patterns were then used to decode the presence of mental states during 10 min of breath-focused meditation, which characterized the unique fluctuation of internal attention states for each meditator. To our knowledge, this task was the first to provide brain-derived estimates of attentional focus during meditation at the individual level, such as the percentage time focused on the breath (Weng et al., 2020). Importantly, this individualized multivariate approach is well-suited to studying diverse populations because it allows for individual variability of neural representations of mental states.

In this paper, we develop and pilot the feasibility of an Intersectional Neuroscience framework to study diverse meditators, which is rooted in an inclusive and equitable approach to conducting contemplative neuroscience using multiple methods. First, we partnered with the diverse East Bay Meditation Center (EBMC) in Oakland, CA using community engagement to create study criteria, procedures, and materials that were culturally sensitive to racial and ethnic minorities, sexual and gender minorities, people with disabilities, and people with lower socioeconomic resources. Next, we conducted the EMBODY task, which was designed to accommodate diverse neural structure and function, with an initial sample of 15 diverse EBMC meditators. We tested whether the individualized nature of the EMBODY task would be feasible in this diverse sample, where machine learning classifiers could be used to (i) recognize participant-specific brain patterns relevant to breath-focused meditation (breath attention, mind wandering, self-referential processing), (ii) decode these mental states that uniquely fluctuate during meditation practice for each meditator, and (iii) quantify individual-level attention metrics such as the percentage time attending to the breath (Weng et al., 2020).

## Materials and Methods

### Intersectional Neuroscience, Approach 1: Community Engagement to Increase Diversity of Participants

**FIGURE 1:**
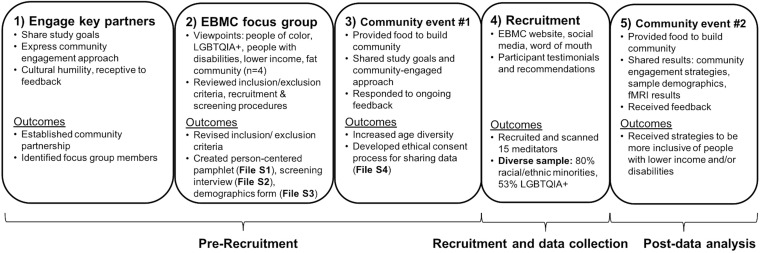
Community engagement steps to increase cultural sensitivity and diversity within fMRI studies. Community engagement with the East Bay Meditation Center (EBMC) was conducted in 5 main steps: (1) engaging key community partners, (2) conducting an EBMC focus group to make study procedures inclusive of people of color, sexual and gender minorities, people with disabilities, and people with lower income, (3) holding a community event at EBMC to share information about the study and receive additional community feedback, (4) recruitment using the EBMC website and social media, and (5) sharing study results at a second community event and receiving additional feedback to improve inclusivity and cultural sensitivity. Steps 1–3 were conducted before study recruitment (∼1 year), Step 4 was conducted during recruitment and data collection (∼2 years), and Step 5 was conducted after data collection and analysis. Main outcomes included revising inclusion/exclusion criteria to be more inclusive, creating person-centered study materials (pamphlet, screening interview, demographics form), developing an ethical consent process for data sharing, and recruiting a diverse sample of EBMC meditators. Key community members received coauthorship on presentations and papers for their work. To conduct community engagement activities, researchers used grant funding to support EBMC for community consultation, space rental, and providing food for events.

In a review of racial and ethnic demographics in U.S. fMRI studies of meditation (SI p. 2; Supplementary Figure S1), we found an under-representation of Black or African Americans, Native Americans or Alaska Natives, Hawaiian or Pacific Islanders, and Hispanic or Latinx people compared to the 2010 U.S. Census. These groups may be under-represented because they have been historically marginalized by scientific communities (Green et al., 1997; Goering et al., 2008). To make our contemplative neuroscience study more diverse and inclusive, we applied CBPR principles (Wallerstein and Duran, 2010) in an academic-community partnership between the University of California San Francisco and the East Bay Meditation Center (EBMC^1^) in Oakland, California. EBMC is considered one of the most diverse contemplative communities in the U.S. (see Footnote 1; 2014 class enrollment included 54% racial/ethnic minorities and 42% LGBTQI+). EBMC was founded on principles of diversity and social action, and provides inclusive contemplative spaces for marginalized groups, such as racial and ethnic minorities, sexual and gender minorities, and people with disabilities (Yang, 2017). EBMC’s culture is centered around social justice, where they have Agreements for Multicultural Interaction^2^ to guide respectful communication among members who vary in identities and social power, and offer year-long courses such as *Practice in Transformative Action* specifically for social justice activists. Thus, EBMC was an ideal community to partner with in making neuroscience procedures more inclusive of diverse meditators. We applied an intersectional framework to improve the study’s inclusivity of people belonging to multiple marginalized identities and received feedback from EBMC in several stages: engaging key community members, conducting a focus group to review neuroscience procedures, holding community events at EBMC to share information about the study, and receiving ongoing community feedback (Figure 1).

#### Engaging Key Community Members

We reached out to two key EBMC community members: Mushim Ikeda, a core teacher and internationally known mindfulness teacher, and Xiaojing Wang, the Programs and Finance Director. We established an initial working relationship through expressing our goals to make our study more inclusive, being open to feedback, and practicing cultural humility (Tervalon and Murray-García, 1998). We directly acknowledged the history of unequal power dynamics between scientists and marginalized groups, and asked for direct feedback if unequal interactions occurred in the study (SI p. 3). Communication was conducted via e-mail, phone, and an in-person meeting in Oakland. Once our team was vetted by the key contacts, they reached out to EBMC members to form a focus group to provide study feedback. Key community partners were paid $40/hour for consultation, and focus group members were paid $30/hour.

#### Focus Group

The focus group was composed of three EBMC members, representing perspectives from the People of Color sangha (racial and ethnic minorities), the Alphabet Sangha (LGBTQIA+ people), and the Every Body Every Mind sangha (people with disabilities and chronic health conditions). In addition, the key contact Mushim Ikeda (MI) was present to facilitate communication and provide feedback. Perspectives were also given to represent the fat community (Dickins et al., 2011) and people with lower income. Food was provided from a local restaurant to build community, and was clearly labeled to avoid allergic reactions. The Principal Investigator (HYW) provided handouts and went through current standard procedures for recruitment, screening, and conducting fMRI scans. Feedback was given in inclusion/exclusion criteria to make the study more inclusive of under-represented groups, to create person-centered recruitment materials (pamphlet with welcoming language that described study requirements, including MRI information), to assess demographics with language representing lived identities as well as standard reporting categories, and to ensure disability access to the MRI scanner.

Importantly, because the study was using individualized neuroscientific methods, inclusion/exclusion criteria could be revisited including current medical and psychiatric illness, as well as substance use. Changes were made as long as they did not interfere with the main purpose of the experiment: to measure attentional states during breath-focused meditation. By including and responding to perspectives from *multiple marginalized identities*, we are embodying principles of Intersectional Neuroscience by ensuring people who have one or more marginalized identities would have access to the study (such as a person of color who is also queer and has a disability).

#### Community Events

Two community events were held at EBMC to engage the broader community. The first event was held to share information and receive feedback about the study and to recruit potential participants. The event was advertised through community word of mouth and EBMC social media, and promotion images and language were vetted by MI and the event coordinator. The event was 2 h long, with food provided 30 min before (meat and vegetarian options, listing ingredients for people with food sensitivities), and ∼30 members attended. The event included an introduction (EBMC staff member) and guided meditation (MI), a slide presentation on the community-engaged neuroscience study with diverse EBMC meditators (HYW), and a group discussion of what EBMC members would like scientists to study.

The second community event was held after the neuroscience study was completed (*N* = 15). We shared preliminary results in the diversity of the sample and the neuroscience findings, and received additional feedback on how to make the study more sensitive to people with lower income and people with disabilities (who were still under-represented in our sample).

#### Recruitment and Screening

Participants were recruited from the community event, EBMC social media postings, and word of mouth through community members. Study team members were trained in culturally sensitive recruitment and screening procedures. Importantly, some EBMC members would check with those who had already participated to see if they had positive experiences with the study team before agreeing to participate. This highlights the importance of having a culturally sensitive team. Participants were phone screened with the revised person-centered interview.

#### Ongoing Community Collaboration and Feedback

Community and participant feedback were welcomed throughout the study. During the community event, an EBMC member from the People of Color sangha (DF) brought up concerns regarding neuroscience data safety and consent procedures, particularly when using participants’ individualized neural signals which could potentially re-identify them. Cultural shifts in scientific practices encourage researchers to openly share partial or full datasets after publication, particularly with “big data” sources such as genetics and neuroimaging data (Lunshof et al., 2008). One key issue in research with marginalized populations is whether data are ethically consented to be used for secondary analyses and shared with other researchers (Goering et al., 2008). In collaboration with DF, we developed a community-engaged ethical consent procedure for participants to consent to sharing different types of data (demographics, survey, fMRI) and to be informed of potential medical issues from the brain scan. We drafted the consent form, sent it to community members for feedback, and received IRB approval.

#### Summary of Community-Engaged Recruitment, Screening, and fMRI Procedures

Based on community feedback, we updated inclusion/exclusion criteria, recruitment and phone screening procedures, demographics assessment, consent procedures for data sharing, and MRI scan procedures to be more culturally sensitive by being more inclusive and person-centered (Table 1). Inclusion/exclusion criteria were made more inclusive in terms of meditation experience requirements (≥5 years of ≥90 min weekly practice over the lifetime, taking into account life experiences and financial constraints), age (25 years and older), and typical health criteria (being inclusive of more mental and physical health conditions as long as they did not impact daily functioning or the main process being studied: attention to breath and meditation). In addition, participants were included if they used drugs or alcohol, as long as they were willing to abstain before the fMRI scan. A study pamphlet was created that used person-centered language to describe the study goals and criteria, groups included, fMRI environment, and potential exclusion criteria (Supplementary File S1). The pamphlet was distributed at EBMC so potential participants could self-select in or out of the study, and the study was also advertised online using EBMC social media. A phone screening interview was updated to reflect these person-centered changes to procedures and language (Supplementary File S2). We updated the demographics questionnaire to assess both lived identities (where participants could self-identify) as well as scientific categories (Supplementary File S3). We created an informed consent procedure and form for sharing data, where participants were informed of the benefits and risks of sharing data, and could choose which data they were willing to share (Supplementary File S4). Finally, MRI procedures were accommodated for comfort and claustrophobia (participants could schedule a 30-min practice session), mobility and vision issues, and head shape and size (64 or 20-channel head coil used; Table 1).

**TABLE 1:** Summary of community-engaged changes to fMRI research procedures to improve inclusivity and cultural sensitivity.

Study Procedure	Changes	Rationale
**Inclusion/Exclusion Criteria**	• Meditation experience: originally 5 consecutive years, with ≥ 2 weeks of silent retreat practice (Weng et al., 2020). Modified to 5 years over the lifetime, with any form of extended practice with teacher guidance (such as a half-day retreat or longer class) • Age: Original range was 25–65 years; modified to 25 years and older • Standard fMRI inclusion/exclusion criteria screen out participants who may have different brain structure and function. Modification: only exclude people who would have difficulty performing the fMRI task, have health conditions that directly impact smooth breathing or ability to focus on daily tasks, or are unwilling to abstain from substances that impact attention before MRI	Allow for breaks in practice due to life events (such as childbirth); silent retreats may require additional financial and time resources Greater age range is more inclusive and feasible with individualized approach Group-normed averaging not required with individualized MVPA, so can include more groups with diverse neural structure and function. Inclusion/exclusion based on fMRI safety, comfort, and ability to do task (pay attention to breath and internal experiences)

**Person-centered study pamphlet (Supplementary File S1)**	Content of the pamphlet: • Used language that reflected the interests of participants (whether the study would be a good fit for them), not the goals of scientists (whether participants are a good fit for the study) • Explicitly stated the study goals of diversity and inclusivity • Described inclusion criteria (meditation practice and age) and participation requirements (study steps, time, and compensation). • Described the fMRI environment and task using images and simple descriptions • Explained exclusion criteria in a person-centered way (“Who cannot currently participate”) and described the rationale for excluding people with conditions that impact attention (schizophrenia and/or bipolar disorder) • Included a map and directions to the study site, public transportation options, and travel resources for people with disabilities	Focus group feedback underscored importance of clearly demonstrating cultural sensitivity and describing the study procedures and inclusion/exclusion criteria in simple language Materials described study using person-centered approach that emphasized the perspective of diverse participants Potential participants were able to self-select in or out based on the main criteria before contacting the study team for a phone screening interview

**Phone Screening (Supplementary File S2)**	• Revised to use person-centered language to assess safety and comfort in the MRI, medical and psychological functioning, pregnancy status for all genders, current substance use, and potential disabilities that could benefit from accommodations • Gender identity and pronouns assessed • Study personnel trained in the person-centered approach and given rationale for changes made based on community feedback	Assess eligibility and study accommodations in a cultural sensitive way Ensure study personnel used appropriate pronouns throughout the study

**Demographics assessment (Supplementary File S3)**	• Participants could self-identify using their own language for race, ethnicity, and gender identity (i.e., *“How do you self-identify your gender?”*) • Contextualized and provided scientific terms from which participants could self-select	Represent both personal and scientific perspectives

**Informed consent procedure for data sharing and incidental medical findings (Supplementary File S4)**	• Developed an additional consent form and procedure for participants to be fully informed of, and consent to, both the benefits and risks associated with sharing their data (demographics, brain, survey) with the scientific community • Participants could indicate whether they wanted to be notified if scientists found a concerning MRI result (while acknowledging this was not a clinical scan) • Participants could choose each type of data they were willing to share from demographics, survey, and brain data. Brain data were described in terms of original raw data and reduced analyzed data, such as individualized brain maps and resulting attention metrics	Ensuring an ethical consent process to share data from under-represented groups, particularly when participants’ individualized neural signals which could potentially re-identify them

**MRI scan procedures**	• Option for participants to have a test MRI visit to assess comfort • Falls prevention training for staff to help participants with mobility issues • Study could accommodate participants who are vision-impaired (auditory instructions used) • To accommodate head shape and size, can use 64- or 20-channel head coil • Study team built relationships with the participants by validating any difficult experiences (e.g., anxiety within the medical environment), made any accommodations requested, and dialogued about EBMC experiences	Cultural sensitivity and skills for those with MRI comfort, movement, and vision issues Individualized MVPA analyses can accommodate differences in equipment Building emotional safety and responsiveness

### Participant Demographics and Diversity

Participants were 15 adults (mean age = 40.0, SD = 7.0, range 26–72), with at least 5 years of lifetime meditation practice (> = 90 min/weekly, where half of practice is on breath and/or body sensations), and at least a half-day of retreat or class practice. To show the range in lived identities, Table 2 reports gender identity, pronouns, race, ethnicity, and sexual orientation using both (1) self-identified and (2) corresponding standardized reporting categories chosen by participants. Gender identities included cisgender male (*n* = 9), cisgender female (*n* = 5), and another identity (queer, *n* = 1). Many participants (80%, 12/15) reported being racial minorities, including 6 participants who reported being multi-racial (40%). 36.4% (4/11) of participants reported their ethnicity as Hispanic or Latino (also called “Latinx” to represent all genders). Participants self-identified a wide range of ethnicities beyond the two NIH categories. Note that some participants identified their race as Latinx, even when NIH separates race and ethnicity into separate categories, showing the diversity of lived experiences. Almost half of the sample reported being a sexual minority (46.7%, 7/15), including 4 participants identifying as Lesbian/Gay/Homosexual, 2 participants identifying as Bisexual/Pansexual, and 1 participant identifying as Asexual. Some participants reported being a member of both a racial/ethnic minority and a gender and/or sexual minority, showing that participants with intersectional identities were represented.

**TABLE 2:** Self-Identified Participant Characteristics with Corresponding Standardized Reporting Categories.

	Self-Identified characteristics (n)	Corresponding standardized reporting categories	Percent (*n*)
**Gender**	Cisgender female (1), female (2), woman (2)	Female	33.3 (5)
	Cisgender male (2), cis male (1), male (6)	Male	60.0 (9)
	Queer (1)	Another identity such as transgender, intersex, and/or non-binary genders	6.7 (1)
**Pronouns**	He/Him/His (8)	Not applicable	53.3 (8)
	She/Her/Hers (5)		33.3 (5)
	They/Them/Theirs (1)		6.7 (1)
	Other: He/Him/They (1)		6.7 (1)
**Race**	African American (1)	Black or African American	6.7 (1)
	Purepecha, Indigenous (1)	American Indian or Alaska Native	6.7 (1)
	Asian American (1), Pilipino-AM (1), South Asian Indian (1), No response (1)	Asian	26.7 (4)
	White (3)	White	20.0 (3)
	African American, White (1); Black (2); Chicano, Native, White (1); Latinx (1); Latinx/Mixed (1)	Multi-racial*	40 (6)
**Ethnicity**	Chicano/Native American and White (1); Iranian (1); Latinx, Chicanx, Halfsican, Queer (1); Purepecha (1)	Hispanic or Latino	26.7 (4)
	African American, Scottish (1); Buddhist (1); Gujarati, Indian, South Asian American (1); Jewish, English (1); Korean American (1); Midwestern (1); Pilipino-American (1), No response (4)	Not Hispanic or Latino	73.3 (11)
**Sexual**	Lesbian (1), Queer (1), No response (2)	Lesbian/Gay/Homosexual	26.7 (4)
**Orientation**	Bisexual (1), Queer (1)	Bisexual/Pansexual	13.3 (2)
	Heterosexual (4), Infrequent (1), No response (2)	Straight/Heterosexual	46.7 (7)
	Queer (1)	Asexual	6.7 (1)
	Queer (1)	Do Not Wish to Specify	6.7 (1)

Using the demographics form developed through community engagement, participants reported their self-identified categories representing their lived experiences for gender, pronouns, race, ethnicity, and sexual orientation. In addition, they chose which corresponding standardized category they wanted to be reported in the scientific literature. Asking for both self-identified and standardized categories improves cultural sensitivity of studies by honoring the lived identifies of participants while also providing information for scientific reporting. Standardized reporting categories for race and ethnicity were based on guidelines from the U.S. National Institutes of Health (NIH). *Participants indicated all NIH categories of races that applied. If they chose more than one race, they were included in the multi-racial category.

In Table 3, additional participant demographics are included to characterize aspects of socioeconomic status (education level, employment status, income, and health insurance) and spoken languages. The sample was highly educated, where all 15 participants received a Bachelor’s degree or higher. Most participants were working for pay (73.3%, 11/15), and the mean and median income were in the range of $50,000-$59,999. 80% of participants had health insurance. All participants spoke English, and 40% spoke additional languages. In addition, 53% of participants (8/15) reported Buddhism as a religious affiliation. Based on the more inclusive criteria, the study also included participants who reported current mental health issues (20%, 3/15), were currently taking psychiatric medications (13.3%, 2/15), or experienced migraines (6.7%, 1/15). No participants reported having any physical disabilities.

**TABLE 3:** Additional participant demographics.

Characteristic	
**Age, mean (SD)**	40.0 (7.02)
Range	26–52

**Education**	**Percent (*n*)**
Bachelor’s degree	20.0 (3)
Some graduate work	13.3 (2)
Master’s degree	53.3 (8)
Doctoral degree	13.3 (2)

**Employment status**	
Working for pay	73.3 (11)
Unemployed and looking for work	6.7 (1)
Student	13.3 (2)

**Income**	
0- $19,999	20.0 (3)
20,000 - $49,999	26.7 (4)
50,000 - $99,999	33.3 (5)
Over $100,000	20.0 (3)

**Has health insurance**	80.0 (12)

**Spoken Languages**	
English only	53.3 (8)
English and other language(s)	40.0 (6)
No response	6.7 (1)

*Additional participant demographics including age, aspects of socioeconomic status (education, employment, income, health insurance), and spoken languages.*

### Intersectional Neuroscience, Approach 2: Individualized Neuroscience Methods to Accommodate Neural Diversity

#### EMBODY Task: Individualized Machine Learning to Measure Mental States During Meditation

The EMBODY (**E**valuating **M**ultivariate Maps of **BODY** Awareness) task uses the strengths of individualized multivariate neuroimaging analytic methods (multi-voxel pattern analysis or MVPA; Norman et al., 2006; Haxby et al., 2014) to accommodate structural and functional neural diversity. The EMBODY framework uses MVPA applied to fMRI data to learn and decode mental states during meditation, producing novel individual-level metrics of internal attention during meditation, such as the percentage time attending to the breath (Weng et al., 2020). The EMBODY framework was initially tested in 16 participants, which confirmed the individualized methods were feasible for most participants (87.5%) and all 8 experienced meditators.

Using the same EMBODY fMRI protocol and data analyses as Weng et al. (2020), this brief description highlights how the idiographic approach of the Intersectional Neuroscience framework produces person-specific attention metrics using individualized brain signals. We tested whether the individualized EMBODY task would be feasible in 15 diverse meditators from EBMC. First, we tested whether MVPA classifiers would be able to recognize individualized brain patterns representing internal attention states relevant for breath-focused meditation (Step 1). Next, we used these learned brain patterns to decode or estimate the mental states during the meditation period for each individual, revealing their unique fluctuation of mental states during meditation (Step 2). Finally, these decoded mental states were quantified into individual-level metrics of attention such as percentage time attending to the breath (Step 3). See Weng et al. (2020) for full methodological details, which are briefly reviewed below.

#### Procedure

Full eligibility was assessed via phone interview (Supplementary File S2). Participants were consented, trained in MRI task procedures, and then completed a 2-h MRI protocol. They were paid $75 for participation and ≤ $20 for travel. All participants provided written informed consent for study participation and data sharing (approved by the Institutional Review Board of the University of California, San Francisco, Protocol #15-16716).

***Step 1: Individualized brain patterns representing internal attention***

**FIGURE 2:**
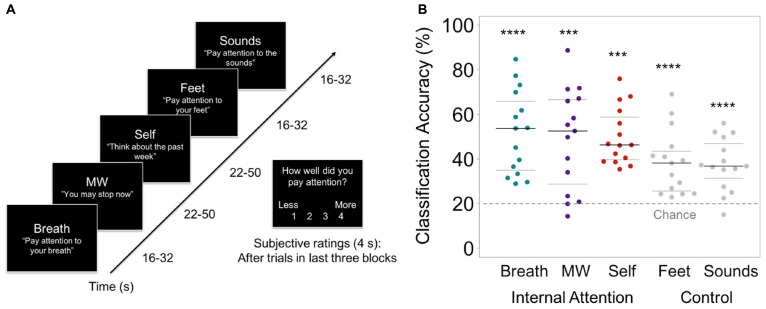
**(A)** Internal Attention (IA) task. With eyes closed, participants were directed via 2-s auditory instructions to pay attention to five internal mental states for brief time periods (16–50s). The IA task directed attention to three mental states relevant for breath meditation (Breath, MW, and Self), and to two control mental states (attention to the Feet [another area of the body] and ambient MRI Sounds [consistent external distractor]). Example auditory instructions are displayed in quotes. MW was induced by instructing participants to stop paying attention and let their minds attend to whatever they wanted. Conditions were randomized over six IA blocks in four orders, with 72s of data collected from each condition in each block (total 432s/condition). For the last half of IA task trials, subjective ratings of attention were collected after each trial (except MW) using a button box (1 = less, 4 = more). **(B)** From the IA task, the prediction accuracy of the classifier for identifying internal states of attending to the Breath, MW, and Self, and control conditions of attending to the Feet and Sounds. Beeswarm plots present each data point, the median (bold black line), and ± 25th percentile range (gray lines) of the mean prediction accuracy for all data in each condition (*n* = 432) across all subjects. Statistical significance was determined by a one-sample two-sided *t*-test against theoretical chance-level for classification of 5 categories (20%, denoted by dashed line). ***ts_14s_< 4.98, *p* < 0.001, ****ts_14_> 5.77, *p*s < 0.0001.

At a participant-specific level, we first tested whether MVPA classifiers could distinguish between fMRI brain patterns of internal attention relevant for meditation in this diverse sample. In the Internal Attention (IA) task, participants were instructed to change their focus of internal attention to five states, including mental states relevant for breath-focused meditation (attention to breath, mind wandering, and self-referential processing) and two control conditions (attention to feet, and ambient sounds; trials ranged from 16 to 50s; Figure 2A). For breath-focus, attention was maintained where they felt the breath most strongly (e.g., nose, throat, chest; see SI p. 4 for full list of breath-related regions). For self-referential processing, participants generated 5 events from the past week, and 5 events that would occur in the next week during the training session. Six blocks were administered from one of four randomized stimulus order sets. See Weng et al. (2020) for full task design and MRI scanning parameters.

**FIGURE 3:**
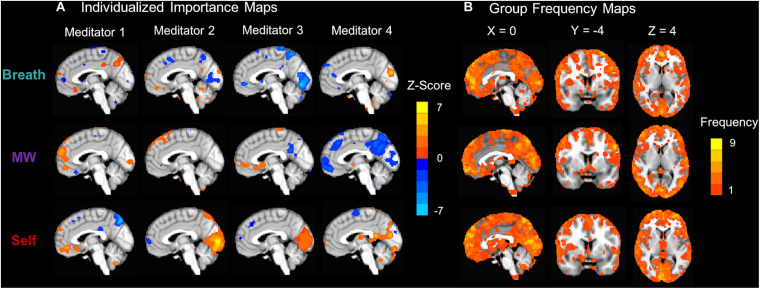
Classifier importance maps representing voxels that accurately distinguish internal mental states. **(A)** Subject-level importance maps showing individualized brain patterns representing voxels that are important for distinguishing neural signatures of attention to the Breath, MW, and Self (*X* = 0). For each task condition, importance values were computed by multiplying each voxel’s classifier weight for predicting the condition and the average activation during the condition (McDuff et al., 2009). The maps were thresholded at ± 2 SD and displayed on the MNI152 template to identify the most important voxels for each participant. Orange importance voxel indicate positive *z*-scored average activation values, and blue importance voxels indicate negative *z*-scored average activation values. **(B)** To examine importance voxels at the group level, group importance frequency maps indicate the number of participants for which the voxel accurately distinguished each mental state. All importance voxels were summed, irrespective of average positive or negative *z*-scored activation. Frequency maps were also computed that independently summed positive (Supplementary Figure S2A) and negative (Supplementary Figure S2B) *z*-scored activation voxels, as well as histograms of frequency counts (Supplementary Figures S2c–e). Note that the maximum frequency for any importance map was 9/15.

To maximize this within-subjects approach, we collected more data within each participant over 6 blocks (2160 brain patterns for classifier training and testing, 432/condition, TR = 1s). Wholebrain MVPA was conducted on pre-processed fMRI data using k-fold cross-validation analysis in native space (penalized logistic regression with L2 regularization and a penalty parameter of 0.01; Weng et al., 2020). Classification accuracy was computed based on the number of correct decisions for each condition (tested against 20% chance levels using a Chi-Square test at the individual level, and with a one-sample *t*-test at the group level). Individuals that showed above-chance accuracy in 2/3 categories for Breath, MW, and Self conditions were used for subsequent analyses including decoding meditation states. Individualized brain patterns representing IA states were computed for each participant using classifier importance maps, which use classifier weight information (thresholded at ±2 SD) to identify which voxels were most important in distinguishing neural patterns of Breath, MW, and Self (McDuff et al., 2009; Weng et al., 2020). To examine how unique vs. common brain patterns were across participants, we computed importance frequency maps, which summed the frequency of participants for which each voxel accurately identified each mental state for: all importance voxels (Figure 3B), positive, and negative importance voxels (see Supplementary Figure S2 for frequency maps and histograms).

To further test whether MVPA classifier accuracy of internal attention was meaningful, we associated accuracy with within-subject subjective ratings of attention for half of the trials (*How well did you pay attention?* 1 = least, 2 = less, 3 = more, 4 = most), which were indicated by button box. Ratings were not administered after MW trials where participants were instructed to stop paying attention. Individual correlations were computed using within-subjects Pearson’s r, and a group-level correlation was tested with Fisher r-to-Z transformation means tested vs. 0. Note that in this study, the ratings instructions were changed from Weng et al. (2020) to make the task easier for participants, which likely decreased the variability of results (See SI p. 6 for description of ratings instructions). See SI p. 4 and Supplementary Table S1 for supplementary analyses controlling for respiration and head motion.

***Step 2: Individualized decoding of meditation period***

By first establishing that MVPA classifiers could reliably distinguish and identify internal attention brain states in Step 1, we could then apply these learned brain patterns to objectively decode (using classifier decisions) the continuous focus of attention using the neural data from a separate 10-min meditation period (600 novel brain patterns). Importantly, this could approach could reveal the fluctuations in attention that are specific to each meditator’s practice session.

***Step 3: Individual-level attention metrics***

We then further analyzed the classifier decisions in Step 3, where we computed novel metrics of attention during meditation, including estimating the percentage time attending to the breath or engaging in mind wandering or self-referential processing, producing novel individual-level attention metrics for each participant. Using classifier decisions, attention metrics were computed for percentage time in each mental state (Breath, MW, Self), number of mental events, mean duration of events, and standard deviation (SD) of events (see Weng et al., 2020 for full details). To preliminarily assess construct validity and inform future research, we characterized the meditation metrics at the group level, and tested whether participants attended longer to breath vs. other mental states during meditation (SI p. 6; Supplementary Figure S3). Data were analyzed in SPSS (v. 24), figures were created with R, and brain maps were displayed using AFNI.

## Results

### Individualized Neuroscience Approach: EMBODY fMRI Task

In our Intersectional Neuroscience framework, we tested whether an individualized approach to fMRI study design and analysis would be feasible in this diverse sample of experienced meditators. We tested whether principles of the EMBODY task (Weng et al., 2020) would be validated in this diverse sample of experienced meditators, which was designed to accommodate diversity in neural structure and function. We tested whether machine learning classifiers could (i) recognize participant-specific brain patterns relevant to breath-focused meditation (breath attention, mind wandering, self-referential processing; Step 1), and (ii) be applied to decode these mental states that uniquely fluctuate during meditation practice for each meditator (Step 2). Finally, these decoded mental states were quantified into individual-level metrics of attention during meditation, such as percentage time attending to the breath (Step 3).

***Step 1: Distinguishing individualized neural patterns of internal attention***

In alignment with an intersectional neuroscience framework, we used individualized brain pattern classifiers to recognize each participant’s internal attention states important for breath-focused meditation based on their unique brain data. This approach accommodates and harnesses diversity in both brain structure and function. Across all participants, each attentional state yielded a distinct neural signature (all classification accuracies > 37% vs. 20% chance for 5 categories, *t*s_14_ > 4.96, *p*s < 0.001; Figure 2B). The brain patterns most relevant for breath meditation were distinguished at more than twice chance levels (breath = 51.8%, mind wandering = 48.9%, self-referential processing = 50.1%; *t*s_14_> 4.95, *p*s < 0.001, Cohen’s *d*s > 1.28), whereas the control conditions were distinguished just below twice chance levels (feet = 38.4%, sounds = 37.6%, *t*s_14_ > 4.98, *p*s < 0.001, Cohen’s *d*s > 1.29; Figure 2B; see Supplementary Table S2 for classifier confusion matrix). Classifier accuracy was not likely driven by respiration rate or head motion between conditions (SI p. 4). Notably, these accuracies were comparable to the first study of the EMBODY task (Weng et al., 2020), and all *t*-tests from both studies showed large effects sizes above 1.0.

The breath meditation-relevant brain patterns were reliably classified in all 15 experienced meditators (where 100% of participants demonstrated at least 2/3 *p*s < 0.001 for breath, mind wandering, and self-referential processing; Supplementary Table S3). All participants were analyzed in subsequent analyses to decode the meditation period. Trial-level classification accuracy was not significantly correlated with subjective ratings of internal attention for all trials (mean *Z* = 0.04, *p* = 0.41) or breath trials only (mean *Z* = 0.07, *p* = 0.48; see SI p. 6 for further discussion).

***Distributed Brain Patterns Contributing to Accurate IA Classification***

Classifier importance maps identified the voxels most important in distinguishing between the attentional states (McDuff et al., 2009), which were distributed throughout the brain and unique for each participant (Figure 3B; Supplementary Figure S2). Group-level importance frequency maps showed that most importance voxels predicted classifier accuracy for only 1–2 participants, demonstrating the distributed nature of the patterns. The maximum frequency for any importance voxel was 9/15 or 60% of the sample.

***Step 2: Decoding the focus of attention during breath meditation***

**FIGURE 4:**
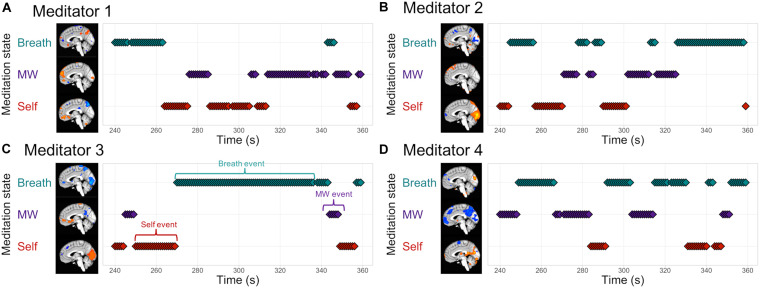
EMBODY Step 2: Decoding the internal focus of attention during breath-focused meditation using individualized brain patterns. Based on each participant’s unique brain signatures for Breath, MW, and Self, classifier decisions were made for each time point of fMRI data (TR = 1s), producing a continuous estimate of attention states during breath meditation. The middle of the meditation period is displayed for four meditators **(A–D)**. Mental events were quantified as 3 or more consecutive decisions from the same mental state **(C)**, and were used to compute metrics of attention during meditation in Step 3.

Individualized brain patterns for each participant were used to decode the focus of attention during 10 min of breath meditation, producing second-by-second decoding of internal attention states of attending to the breath, mind wandering, or self-referential processing (Figures 4A–D). From these data, “mental events” were defined whenever there were 3 or more consecutive time points that were classified as belonging to the same mental state (Figure 4B). Note that each participant’s meditation session is unique in the durations of and fluctuations between mental states.

***Step 3: Quantifying metrics of internal attention during breath meditation***

**FIGURE 5:**
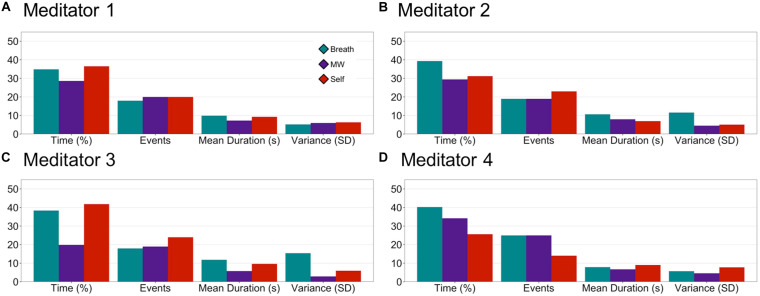
EMBODY Step 3: Individual-level attention profiles during the meditation period. Based on the mental-state estimates during meditation from Step 2, internal attention metrics were quantified for each individual meditator: percentage time spent in each mental state (Breath, MW, or Self), the number of events, mean duration of events (s), and variability (standard deviation or SD) of duration of events **(A–D)**.

Based on MVPA classification of mental states during meditation from Step 2, we computed novel metrics of attention during meditation for each participant, including *percentage time spent engaged* in each mental state, *number of mental events* (or discrete periods engaged in each mental state), the *duration of each mental event*, and the *variance of the durations* (SD). See Figure 5 for individual-level plots of attention metrics during the meditation period. This demonstrated the feasibility of producing fine-grained metrics of attention during meditation, such as estimating the interoceptive focus on the breath, that is specific for each individual’s meditation practice and based on their unique brain signals. For preliminary characterization of attention metrics at the group level, see SI p. 6, Supplementary Figure S3, and Supplementary Table S4.

## Discussion

We developed an *Intersectional Neuroscience* framework in a study of breath-focused meditation, which applies an intersectional lens to neuroscience research to improve inclusion of diverse participants, particularly those who belong to multiple marginalized identities. Within the context of contemplative neuroscience, we increased diversity of participants through employing 1) community engagement to partner with a diverse meditation community, and 2) individualized multivariate neuroscience methods which preserve and harness neural diversity as a statistical strength. By forming a community partnership with EBMC, we adjusted our research materials and procedures to be more inclusive of racial and ethnic minorities, sexual and gender minorities, people with disabilities, people with lower education and income, older adults, and those with mental health and neurological disorders. These changes were successful in recruiting a more diverse sample for the neuroscience study, where 80% of participants were racial and/or ethnic minorities (75% racial minorities, 26.7% Latinx), and 53.3% identified as LGBTIA+. In addition, some participants identified as both racial and/or ethnic minorities and LGBTQIA+, demonstrating the feasibility of using an intersectional approach to increase inclusion of participants who belong to multiple marginalized identities. Further, we established initial feasibility of using individualized neuroscience methods in this diverse sample, and found that MVPA classifiers in the EMBODY task could recognize unique brain patterns representing internal attention states in every meditator. This individualized brain patterns could then be used to estimate the fluctuating mental states during each meditator’s meditation session, and produce individual-level metrics of attention during meditation.

Community engagement involved continuous dialogue between scientists and EBMC members, as well as iterative revision of study materials and procedures. We revised inclusion and exclusion criteria to be as inclusive as possible, used person-centered language from the perspective of participants, and developed an ethical consent process to sharing data. Among the person-centered changes, we adjusted the way we assessed participant demographics by asking for their lived identities *first* and then the standardized reporting categories (for gender identity, race, ethnicity, and sexual orientation). This structure communicates that their personhood and lived experiences are valued foremost, and then standardized reporting categories are assessed for scientific purposes (acknowledging that science operates within a broader cultural context). This way of assessing demographics can be more welcoming and inclusive to a broader range of people, and allow them to indicate identities that may not be included in standard demographic forms, such as transgender or non-binary gender identities. For the purposes of this paper, we reported both lived identities and chosen corresponding standardized reporting categories to show the full range of diversity. We recommend assessing and reporting a broader range of identity demographics (such as sexual orientation and disability status, even if those groups are not the main focus of the study) so that people who belong to under-represented groups can assess whether study findings may generalize to them.

Using community-engaged methods was successful in recruiting a more diverse sample (*N* = 15). Our sample included members of under-represented groups in the neuroscience of meditation (see SI p. 2, Supplementary Figure S1), including Black or African Americans (when including multiracial people: *n* = 4 or 26.7%), Native Americans (when including multiracial people: *n* = 2 or 13.3%), and Hispanic or Latinx people (*n* = 4 or 26.7%). Our study also included Asian Americans (4 or 26.7%) and White people (3 or 20%). Further work is needed to be more inclusive of groups not represented such as Hawaiian or Pacific Islanders. Our fMRI demographics review also showed that only two gender identities were included (male, female), and our study included and assessed more gender identities such as transgender, intersex, and/or non-binary genders (*n* = 1 or 6.7%). Overall, the study had greater LGBTQIA+ representation with 53% of participants identifying as a gender or sexual orientation minority (including those identifying as lesbian/gay/homosexual, bisexual/pansexual, and asexual). Although the study was more diverse in some demographics (race/ethnicity, sexuality, and gender identity), it lacked diversity in socioeconomic status and disability status. Community feedback from EBMC included doing outreach at community centers that serve lower-income people, and advertising cash payment and travel reimbursement more clearly. Further outreach may be needed to be more inclusive of people with disabilities, and to those who are not comfortable within an academic medical environment (at least one person chose not to participate for this reason). Our study could be more inclusive of the fat community by using neuroscience modalities that are less restrictive in head and body size, such as electroencephalography. Person-centered recruitment and screening materials developed with EBMC are included in the SI, so that researchers may adapt these materials for their populations of interest (ideally using community engagement and focus groups).

Another way we made our study more inclusive was using individualized multivariate neuroscientific methods, which preserve each person’s diversity in brain structure and function. This allowed us to revise inclusion/exclusion criteria to be much more inclusive of groups who are considered to have “non-normal” brains such as individuals with mental health or neurological disorders, those who take psychotropic medications, and older adults. This is particularly important within contemplative neuroscience because people often learn and practice meditation as a non-pharmacological approach to manage mental and/or physical health symptoms. By shifting the main neuroscientific questions to asking which *mental states* are present during meditation (which are estimated by brain patterns), rather than which *brain regions* are being activated averaged across the group, we can harness the power of individual neural diversity to understand what is going on internally during meditation for each participant.

We tested whether principles of the EMBODY task, an fMRI task designed to accommodate and harness neural diversity using individualized machine learning methods, would be validated in this diverse EBMC sample. First, we demonstrated that for all 15 diverse meditators, machine learning classifiers recognized individualized brain patterns representing internal attention states relevant for breath-focused meditation (attention to breath, mind wandering, and self-referential processing). Even with no external changes in visual stimuli (eyes remained closed during the experiment), when meditators were instructed to direct their attention to different internal stimuli, these brain patterns were consistent enough to be recognized by individualized machine learning classifiers. However, compared to the first study of the EMBODY task (Weng et al., 2020), we did not find significant within-subject associations between subjective ratings and classifier accuracy of IA states in this study. Participants in the first study reported difficulty in using all four ratings, so in the current study, we de-emphasized the need for using all ratings, which likely decreased power to detect any associations. In addition, experienced meditators often reported having the highest level of attention for many trials, and may not be aware of more subtle changes in attention that MVPA classifiers may be able to detect. This study was also more inclusive of people who may have varying levels of attention or ability to report on attention, with participants reporting a current mental health issue, taking psychiatric medications, or experiencing migraines. Future research may improve methods for assessing ratings, such as using a dial which would have more variability, and examining whether certain groups that show differences in attention may perform differently in the task.

Brain patterns representing internal attention states of attention to breath, mind wandering, and self-referential processing were unique for each meditator. This was highlighted by group-level frequency data where the majority of importance voxels across the brain contributed to accurate classifier accuracy for only 1–2 participants (Figure 3B; Supplementary Figure S2). The maximum number of participants that shared a single importance voxel for any mental state was 9 (out of *N* = 15 or 60%). This demonstrated that no importance voxels were shared across the entire sample, which highlights the variability and diversity of neural patterns. However, these findings came from using an initial thresholding procedure to identify importance voxels, and more research is needed to understand the potential sources of variability in neural patterns. For example, when participants focus on the breath, variability in brain patterns may be related to the different locations in the body where attention was placed (e.g., nose, chest, or stomach; see SI p. 4 for list of regions). Accurate classification for different individuals may also be due to physiological factors such as differences in respiration and heart rate. Further, analyzing importance voxels in different ways may yield different results and insights into brain function involved in internal attention. Rather than thresholding to find the most variable and extreme voxels within each participant, a lower threshold range may be chosen (±1.0 to 2.0 SD) which may identify a larger number of more modestly important voxels. This method may identify more voxels that are common across participants, which can potentially serve as voxels for group-level classifier training. Alternate methods that can quantify the influence of each voxel include bootstrapping methods that selectively remove each voxel and recompute classification, and calculating the univariate effect size of each individual voxel contributing to the overall multivariate result (McArtor et al., 2017).

We also demonstrated the feasibility of using these unique brain patterns to decode or estimate the fluctuating focus of attention during 10 min of breath meditation. This approach made the invisible processes of meditation more visible, and revealed that each meditator experienced a different pattern of fluctuation between mental states of attention to breath, mind wandering, and self-referential processing. These decoded mental states could then be quantified into metrics of internal attention during meditation: percentage time attending, number of events, and mean duration and variability of events. Using these metrics, attention profiles could be computed for each individual, showing the feasibility of using individualized brain patterns to estimate subject-level attention metrics. Participants varied in how their attention fluctuated during meditation, and in the resulting pattern of attention metrics such as percentage time attending to breath, mind wandering, or self-referential processing. This study was designed to estimate mental states during meditation without interfering with the meditation process by requiring subjective or motor responses (Levinson et al., 2014). Future research should further validate the presence of mental states during meditation through validation tasks that compare classifier decisions of mental states with meditator self-report (Hasenkamp et al., 2012), as well as compare attention profiles between meditative vs. non-meditative states (such as resting state) or pre vs. post-meditation training.

These results were mostly consistent with the first EMBODY task study, which was conducted with a less racially and ethnically diverse sample without community engagement methods (Weng et al., 2020). Together, these two studies showed that individualized machine learning classifiers could recognize internal attention states for most participants (*N* = 29/31 or 93.5%), and all experienced meditators studied thus far (*n* = 23). By collecting more data within each participant to identify subject-level effects, which is common for within-subjects study designs (Smith and Little, 2018), each person’s brain can be treated as its own unique environment and serve as its own statistical baseline. This approach seems feasible particularly in experienced meditators, who have spent more time attending to internal states, and may therefore have more stable neural patterns that can be recognized by MVPA. Further research is needed to see how feasible the approach is in novice control participants, who are the majority of people included in clinical trials of mindfulness and meditation (MVPA recognized brain patterns in 6/8 or 75% of controls [Weng et al., 2020]). Alongside the individualized approach, group-level classifiers may be built with larger samples to help detect mental states in novice controls, particularly those whose unique signals cannot yet be detected with small samples of data (Haxby et al., 2011; Chen et al., 2017). Both studies also showed that fluctuations of mental states during the 10-min meditation period varied by each meditator, and could be quantified to produce metrics of internal attention during meditation.

Although these metrics are produced using unique brain data, with larger samples they can be combined at the group level to investigate overall patterns in attention during meditation. This bridges the gap between individualized metrics and group-level data analysis, where attention metrics *derived* from the individual-level brain data are the values being averaged and analyzed at the group level, not the brain patterns themselves. By analyzing metrics at the group level, scientists can begin testing whether attention to breath is greater vs. other states during meditation, whether experienced meditators and novices differ in their attention to breath, and test whether individual difference factors influence attention to breath (such as lifetime meditation practice or trait mindfulness). However, much larger samples are needed to test these questions, and by improving inclusivity of neuroscience research, results from more diverse studies may be more generalizable.

For this particular study, our goal was to make the study as inclusive for diverse participants as possible, while maintaining the scientific purpose of studying breath-focused meditation. We included people from a functional standpoint – as long as they could pay attention to internal experiences and were safe and comfortable in the scanner, they were included. The more inclusive approach suggests the resulting attention metrics may be influenced by various factors such as meditation experience level, mental health conditions, taking psychotropic medications, and socioeconomic status. Future research may investigate differences in attention metrics depending on group status with larger sample sizes. For this study, we demonstrated participants do not need to be excluded based on having different brain structure and function in and of itself, particularly because individualized neuroscience methods decrease the need to find group-averaged results. Any neuroscience method that can be conducted at the individual level in native space may be used within an Intersectional Neuroscience framework, including functional connectivity (Finn et al., 2015; Poldrack et al., 2015) and representational similarity analysis (Kriegeskorte et al., 2006). This suggests that future studies, depending on their specific goals, may be more thoughtful about inclusion and exclusion criteria, with particular attention brought to exclusion of multiple marginalized groups. More inclusive studies will increase generalizability of results and may potentially translate to more groups.

The EMBODY framework may be a feasible approach to harness individual brain variability and produce more fine-grained metrics of how attention is deployed during meditation. This approach may be extended to measure mental states during other types of meditation, such as empathic care and distress (Ashar et al., 2017) during compassion meditation. With better measures of how attention is cultivated via meditation, we may better understand what skills are being learned by each meditator, and how these skills may contribute to improved well-being at individual and population levels. In summary, we have outlined a framework to conduct contemplative neuroscience from an Intersectional Neuroscience perspective, particularly using the methodologies of community engagement and individualized neuroscience techniques. This framework is rooted in the compassionate goal to include and empower under-represented groups, particularly those who belong to multiple marginalized identities. Importantly, this framework is not static and can be updated to include more groups and research methods. The framework thrives from continuous dialogue with the community, and counters the *status quo* in academia where knowledge is transmitted one-way from researchers to the community. We have continuously learned from EBMC how to make the study more diverse and inclusive. Academic communities have also learned how EBMC has built their multicultural community to practice meditation and engage in social action, and have adapted their Agreements for Multicultural Interactions for their own organizations (e.g., UCSF Osher Center for Integrative Medicine, Mind and Life Institute). Researchers have also given back to the EBMC community through paying consultants and participants, holding events at EBMC, and sharing EBMC’s culture at academic conferences. This reciprocal exchange of knowledge and resources has the potential to redress power inequities while enhancing inclusivity, quality of scientific methods and findings, and speed and innovation of ideas. This intersectional approach may therefore benefit other fields of psychology and neuroscience, to better understand the groups that are being studied, discover novel insights by actively including them in the research process, and producing more generalizable findings. This framework may also be extended to adopt more principles of community engaged research, where members of the community are included within the research study team, and structural changes are made to improve pipelines in academic research settings (Wallerstein and Duran, 2010). The approach may also benefit from including participants’ lived experiences in a systematic way using qualitative interviewing and focus group methods.

Finally, neuroscience may be used as a powerful tool to bring the public’s attention to areas of societal inequity, and highlight ways to improve social functioning through interventional studies that examine the capacity for social neuroplasticity (Weng et al., 2013). Neuroplasticity may be better measured using individualized multivariate methods, as each person’s unique brain patterns can serve as their own baseline. Individualized approaches may be adopted by more fields of neuroscience to enhance measurement power and increase inclusivity of neuro-diverse participants. To better implement principles of Intersectional Neuroscience, researchers may need to examine their own social identities and privileges, and improve mindful listening and cultural humility skills (Magee, 2019; Yang, 2017). By employing personal reflection and inclusive research methods, the research process has the potential to become an embodied form of compassionate social action that can begin to heal divisions between groups and produce better co-created solutions for well-being and health.

## Data Availability Statement

Code for the EMBODY Task, MVPA analysis, and post-processing are available at https://github.com/HelenWengPhD/embodystudy. MRI data are available from participants who consented to share raw brain data (links at Github page).

## Ethics Statement

The protocol studies involving human participants were reviewed and approved by University of California, San Francisco Institutional Review Board. The patients/participants provided their written informed consent to participate in this study. Written informed consent was obtained from the individual(s) for the publication of any potentially identifiable images or data included in this article.

## Author Contributions

HW, MI, MC, and FH developed community engagement strategy. HW and DF developed the ethical consent process for sharing data. HW and JL-P designed the fMRI task. All authors contributed to the data analytic strategy and interpretation. HW, SS, and VG contributed to data collection, processing, and analysis. HW, JL-P, SS, and VG developed unpublished data analysis tools. HW wrote the manuscript, with contributions from MC and JL-P and comments from all other authors.

## Conflict of Interest

The authors declare that the research was conducted in the absence of any commercial or financial relationships that could be construed as a potential conflict of interest.

## Footnotes

1 https://eastbaymeditation.org/

2 http://www.emergingsf.org/wp-content/uploads/2017/08/EBMC_AgreemntsMulticulturalInteractions15.09.13-copy.pdf
