# Corrigendum: Toward a Compassionate Intersectional Neuroscience: Increasing Diversity and Equity in Contemplative Neuroscience

**DOI:** 10.3389/fpsyg.2020.631816

**Published:** 2021-01-06

**Authors:** Helen Y. Weng, Mushim P. Ikeda, Jarrod A. Lewis-Peacock, Maria T. Chao, Duana Fullwiley, Vierka Goldman, Sasha Skinner, Larissa G. Duncan, Adam Gazzaley, Frederick M. Hecht

**Affiliations:** ^1^Osher Center for Integrative Medicine, University of California, San Francisco, San Francisco, CA, United States; ^2^Neuroscape Center, University of California, San Francisco, San Francisco, CA, United States; ^3^Department of Psychiatry and Behavioral Sciences, University of California, San Francisco, San Francisco, CA, United States; ^4^East Bay Meditation Center, Oakland, CA, United States; ^5^Department of Psychology, University of Texas at Austin, Austin, TX, United States; ^6^Division of General Internal Medicine, University of California, San Francisco, San Francisco, CA, United States; ^7^Department of Anthropology, Stanford University, Palo Alto, CA, United States; ^8^School of Human Ecology and Center for Healthy Minds, University of Wisconsin–Madison, Madison, WI, United States

**Keywords:** meditation, interoception, neuroscience, diversity, community engagement, intersectionality, mindfulness, machine learning

In the original article, there was a mistake in Figure 2 as published. The conditions were labeled incorrectly in Figure 2B. The corrected Figure 2 appears below.

**Figure 2:**
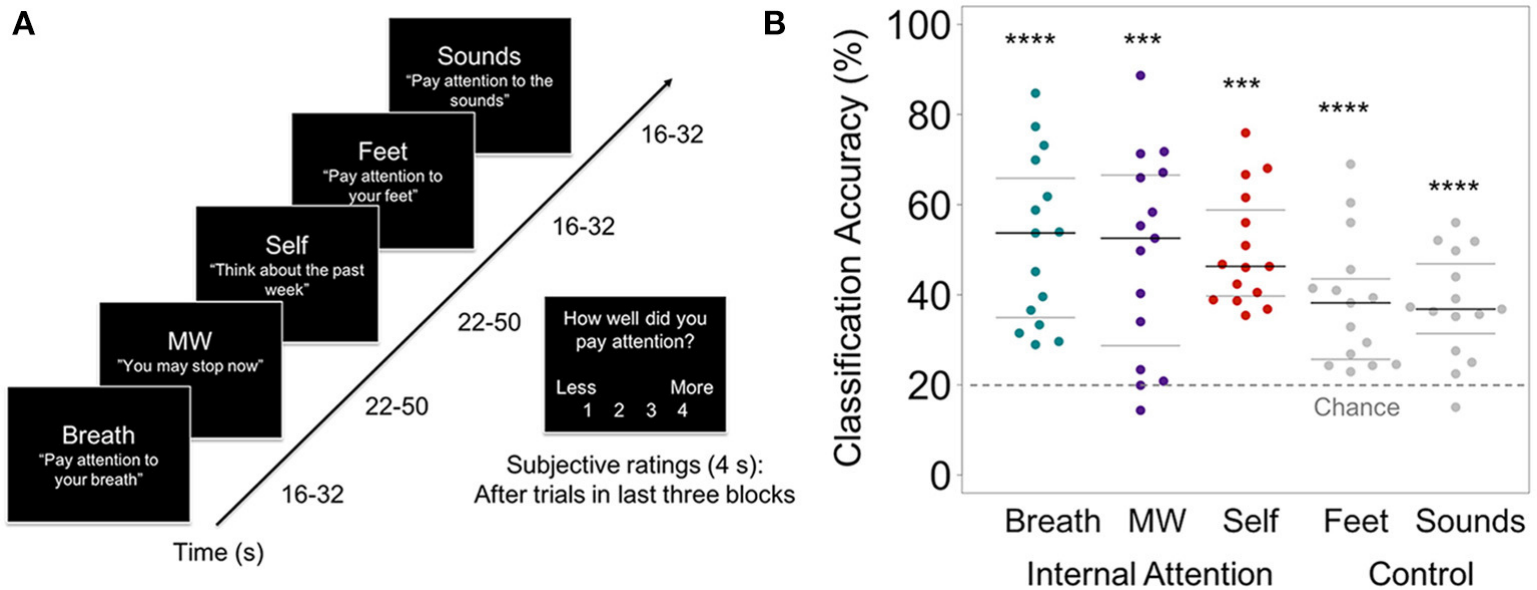
**(A)** Internal Attention (IA) task. With eyes closed, participants were directed via 2-s auditory instructions to pay attention to five internal mental states for brief time periods (16–50s). The IA task directed attention to three mental states relevant for breath meditation (Breath, MW, and Self), and to two control mental states (attention to the Feet [another area of the body] and ambient MRI Sounds [consistent external distractor]). Example auditory instructions are displayed in quotes. MW was induced by instructing participants to stop paying attention and let their minds attend to whatever they wanted. Conditions were randomized over six IA blocks in four orders, with 72s of data collected from each condition in each block (total 432s/condition). For the last half of IA task trials, subjective ratings of attention were collected after each trial (except MW) using a button box (1 = less, 4 = more). **(B)** From the IA task, the prediction accuracy of the classifier for identifying internal states of attending to the Breath, MW, and Self, and control conditions of attending to the Feet and Sounds. Beeswarm plots present each data point, the median (bold black line), and ± 25th percentile range (gray lines) of the mean prediction accuracy for all data in each condition (*n* = 432) across all subjects. Statistical significance was determined by a one-sample two-sided *t*-test against theoretical chance-level for classification of 5 categories (20%, denoted by dashed line). ^***^ts_14s_ < 4.98, *p* < 0.001, ^****^ts_14_ > 5.77, *p*s < 0.0001.

The authors apologize for this error and state that this does not change the scientific conclusions of the article. The original article has been updated.

