# Supplementary material for: Toward a Compassionate Intersectional Neuroscience: Increasing Diversity and Equity in Contemplative Neuroscience
Source: Front Psychol. 2020 Nov 19;11:573134. doi: 10.3389/fpsyg.2020.573134 (PMC7711109; doi:10.3389/fpsyg.2020.573134)
Supplement: Supplementary file 1 [file Data_Sheet_1.PDF]

Who cannot currently participate

People who are:

- **Uncomfortable in MRI scanner environment**
  - MRI size and shape, sensitive to loud sounds, claustrophobic
- **Unsafe to enter the MRI scanner**
  - Irremovable internal or external magnetic devices (pacemaker, pins, screws, external monitors)
  - Irremovable body piercings
- **Unable to lie still for 2 hours**
  - Conditions that cause uncontrollable movement
  - Conditions that impact smooth breathing (asthma, coughing)
- **Having difficulty paying attention to experiences**
  - Difficulty paying attention in daily life (life circumstances, symptoms that impact attention**)
  - Use of medications that impact attention (past 24 hours)
  - Use of recreational drugs (past week)

*** People with schizophrenia and/or bipolar disorder show differences in attention to visual stimuli and will not be included in the current study. This brain task is under development and we will work to make it more inclusive in the future.*

Please give us any feedback about how we can be more culturally sensitive!

embodystudy@ucsf.edu

Directions to Mission Bay

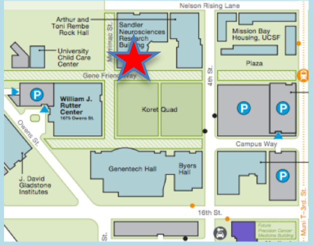

UCSF Neuroscape Lab:
675 Nelson Rising Ln
SF Mission Bay campus

Public Transit

BART 16th St Mission -> free UCSF shuttle to Mission Bay (wheelchair accessible)
BART Powell Street -> SF Muni T line to 3rd St.

Driving by car

From the East Bay
-Take I-80 to San Francisco
-Take exit 2B on the left for Harrison St.
-Turn Right onto Harrison St.
-Turn Right onto Embarcadero
-Continue onto King St.
-Turn Left onto 3rd St.
-Turn Right into UCSF parking garage
-The Neuroscience building is located west towards the Koret Quad just past 4th St. at at 675 Nelson Rising Lane

From the North
-Take US-101 South towards SF
-Take a slight left onto Lombard St.
-Turn right onto Gough St.
-Turn left onto Broadway
-Turn right onto Embarcadero
-Continue onto King St.
-Turn Left onto 3rd St.
-Turn Right into UCSF parking garage
-The Neuroscience building is located west towards the Koret Quad just past 4th St. at at 675 Nelson Rising Lane

From the South
-I-280 N
-As I-280 ends continue onto King St.
-Turn Right onto 4th St.
-Turn Left into UCSF parking garage
-The Neuroscience building is located west towards the Koret Quad just past 4th St. at 675 Nelson Rising Lane

Parking validation available

Parking

To find parking go to 3rd Street Garage located on Campus Way, between 3rd and 4th St.

For participants with disabilities

You may park in the NCRU disabled person parking spaces on Nelson Rising Ln if you:

1. Have a disabled person parking placard
2. Check in with the NCRU front desk and receive a temporary paper parking permit
3. Place the paper parking permit on your car's dashboard

Travel reimbursed on-site up to $20

EMBODY

An inclusive meditation neuroscience study

EBMC

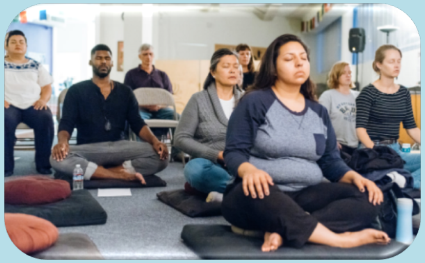

Have experience meditating?

Want to help increase the diversity of meditators within neuroscience studies?

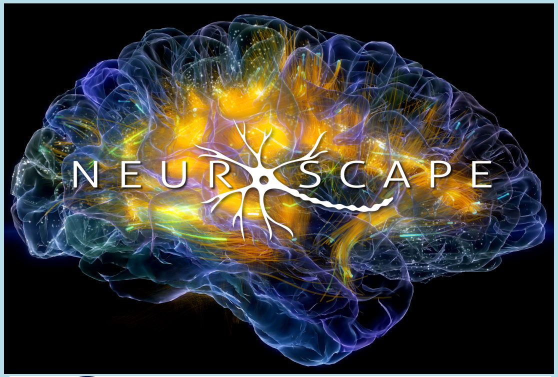

UCSF Osher Center for Integrative Medicine

For more information and to see if you qualify:
embodystudy@ucsf.edu
(415) 514 8445

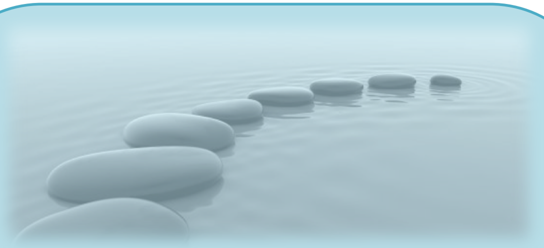

What is the EMBODY Study?

- **Community engagement and dialogue** between EBMC and UCSF to
  - **increase diversity** of meditators in neuroscience studies
  - develop an **inclusive** and **culturally sensitive** approach for

People of Color
LGBTQI+ community
People with disabilities
Fat community

People of all genders are encouraged

- A new brain imaging approach to measure **your unique brain activity** to identify different mental states during breath meditation

We are looking to include Meditators who:

- Have a consistent meditation practice for the past year, and 5 years lifetime practice
  - Age 25 or older
  - Weekly practice, at least 90 min/week (such as ~12 min/day or three 30-min sessions)
  - Includes attention to breath and body
  - Some experience with longer practice (like an EBMC course, retreat)

This study has received feedback from EBMC community members

What would participation involve?

1	45-60 min phone interview<br>30-45 min online surveys
2	3-hour visit to UCSF Mission Bay<br>• 1 hour consent and instructions<br>• 2 hours in brain scanner<br>*Travel options available for people with disabilities*
3	$75 cash payment, travel reimbursement & picture of your brain

What is an fMRI scan like?

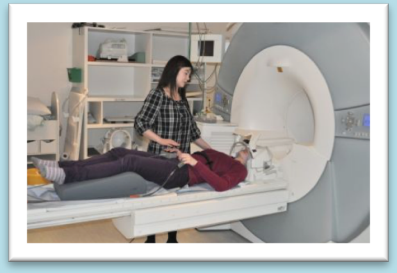

- **Functional magnetic resonance imaging (fMRI)**
  - Uses **magnetic energy** to measure oxygen levels in the brain (**not safe for people with magnetic implants** like pacemaker)
  - Scanner is an enclosed tube
    - widest section is 3 feet (60 cm)
  - Head mask over the face with mirror
  - Scans last 5-8 min long, ~12 total scans
- Requires **2 hours lying still**
- Scanner makes **loud sounds**
  - **We will ensure your comfort** using pillows, pads, blankets, ear plugs etc.
  - Can speak to the scientists between scans
  - Press a squeeze ball at any time to stop

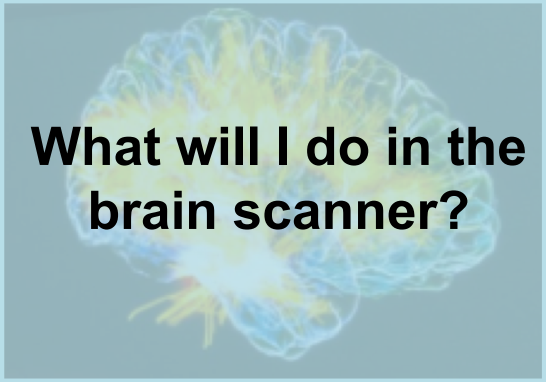

What will I do in the brain scanner?

- Pictures taken of the brain
- Eyes closed or open
- Pay attention to
  - Body sensations
  - Thoughts
  - Sounds
- Look at letters on a screen
- Make responses by pressing buttons

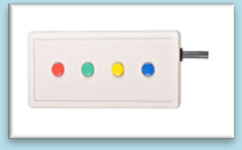

- Heart and breathing rate monitored with sensors

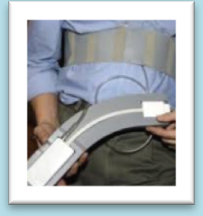

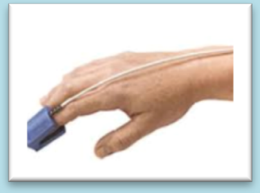

You may stop the experiment at any time
