# Supplementary material for: Toward a Compassionate Intersectional Neuroscience: Increasing Diversity and Equity in Contemplative Neuroscience
Source: Front Psychol. 2020 Nov 19;11:573134. doi: 10.3389/fpsyg.2020.573134 (PMC7711109; doi:10.3389/fpsyg.2020.573134)
Supplement: Supplementary file 2 [file Data_Sheet_2.PDF]

Date: ____ / ____ / ____ Interviewer: _______________

Goal of phone screening: To determine whether the participant is a good fit for the study:

- The pamphlet and EBMC community event should help people determine whether they would be comfortable in the study
- Meditation experience fits criteria
- Study procedures and environment are comfortable and safe
- Participant can complete tasks in scanner
- Aware of any logistical issues and accommodations needed

EMBODY EBMC Study Phone Screening

Name:

Phone:

Email:

Verbal Informed Consent

Hello ________. My name is _________, and I am calling you to follow up about the UCSF Body Awareness study with EBMC. We are contacting you because you expressed interest in the study. Do you have 1 hour to speak with me now? (If not, schedule another time to call)

Great. UCSF researchers from the Osher Center for Integrative Medicine and the Neuroscape Center are conducting a research study to develop a new measure of body awareness using functional magnetic resonance imaging (fMRI). The purpose of this phone call is to tell you about the study and ask you some more questions to determine if you are eligible to participate. We are also working on our procedures to be culturally sensitive for meditators at EBMC. Please do not hesitate to give us feedback in any part of the study in how we can improve our procedures.

We will give you more information about the study, and ask you some questions to see if the study would be a good fit for you. We will ask questions about your age, whether it is safe for you to participate in an MRI study, your health, and experience in mind-body practices. Your answers to these questions will be kept confidential and will not be shared with anyone outside this research study. Answering these questions is completely voluntary, and you can choose not to answer any question that makes you uncomfortable. You may request to stop the interview at any time. Please also let us know if there are ways we could improve this interview, for example the language we use.

Participating in this screening process does include the risk that confidential information you share with the research team could be obtained by persons outside

our research group. The research team will take steps to make as sure as possible that this does not happen. But the possibility of it happening still exists.

If you have any questions about your rights as a participant in this screening process, or you wish to report problems relating to this screening, you may contact the UCSF Institutional Review Board at 415- 476-1814. Please let us know whether your personal information can be written down and studied by the research team to determine whether you may be an appropriate candidate for our study.

Participant _____ Agree (verbally) ______ Disagree (verbally)

About the study:

This study is developing a new measure using fMRI to be able to identify when people are paying attention to body sensations and when they are thinking about something else. The long-term goal of our study is to understand how mind-body interventions such as meditation and yoga can impact body awareness. But for this study, we are only developing this brain measure. We are also working on our procedures to be culturally sensitive for meditators at EBMC and are open to any feedback about the study.

Participation in the study would involve filling out surveys online (about 45 minutes), and making a visit to the Neuroscape Center at the UCSF Mission Bay campus for 3 hours. We will prepare you for the MRI scan by training you in the tasks and making sure you are comfortable in the scanner. You will do tasks such as paying attention to sensations in your body, sounds in the scanner, and internal thoughts; thinking about past, future, and current events in your life; performing a visual task and making button press ratings. We will ask you about your experience after the experiment to refine experimental instructions and procedures. It will take about an hour to prepare you for the scan, and up to 2 hours of being in the MRI scanner. You will be paid with $75 and reimbursed up to $20 for travel costs, in cash which will be paid on-site. We will give you more information about these procedures after asking you some questions. Do you have any questions before we begin? (Answer any questions)

Eligibility Questions:

First, we have some basic questions

1. What is your age? (People who are 25-65 years old are eligible for the study) _____ years

2. What gender do you identify with?
    a. Transgender Woman
    b. Transgender Man
    c. Non-binary: ___________________

d. Woman
e. Man
f. Intersex
g. Another gender: ___________
h. Do not wish to identify

3. What pronouns do you use?
   a. She/Her/Hers
   b. He/Him/Hers
   c. They/Them/Theirs
   d. Other pronouns: ________________

Assess meditation practice

Now I'll ask you some questions about your mind-body practice.

4. What mind-body practice(s) do you practice (such as meditation, yoga, tai chi, Qigong, or any others)? (list all) ___________

5. Are you affiliated, with any practice centers other than EBMC? If yes, please list ____________________________________

For how many years have you been regularly practicing meditation?	less than 5 more than 5 _______ years<br>*5 years lifetime, not necessary to be in one stretch*<br>*stop if less than 5*
Over the last 5 years, within a typical week of meditation practice, how many minutes a week do you meditate?	less than 90 more than 90 ________ min<br>*stop if less than 90*<br>*(approximately 3 30 min sessions or 15min daily)*
In the past year, within a typical week, how many minutes do you meditate?	less than 90 more than 90 ________ min<br>*stop if less than 90*<br>*(approximately 3 30 min sessions or 15min daily)*
What percentage of those minutes are spent in practices that involve attention to body sensations as your main focus, such as to the breath, parts of the body, visceral sensations, and musculature? (e.g., breath meditation, body scan, mindfulness of body-based emotions, walking meditation)	less than 50 more than 50<br>*stop if less than 50*

In the past 5 years, have you engaged in any intensive meditation practice? (such as participation in a day long meditation retreat at EBMC or another meditation center, or participation in a year-long intensive course at EBMC)	Yes No *stop if No*

Assess comfort in the MRI scanner and claustrophobia

Now I'm going to describe the MRI procedure to you in more detail. Please ask me any questions you may have at any time.

The MRI procedure overall will take about 3 hours. First, we will give you instructions and prepare you for the scan for about 1 hour. Then we will set you up to go into the scanner and take pictures of your brain for about 90-120 minutes. Afterwards, we will ask you some questions about your experience.

Now, I am going to ask you some questions to make sure you would be comfortable and safe completing the study procedures. Please let us know if anything concerns you about your comfort and safety for the MRI scan.

The MRI is a large long tube that uses magnetic energy to take pictures of your brain. You will need to lie still on your back inside the tube in order for us to take pictures of the brain, and it makes loud sounds as it takes pictures. At its widest, the MRI tube is 3 feet wide, and for some people this means their arms can be leaning against the MRI, and we can provide padding. For some people, the MRI could be very uncomfortable due to its size and shape. Some people may feel anxious or claustrophobic because the tube is narrow.

You will also wear a head mask that can be quite close to your face. You will mostly have your eyes closed in the experiment, but some tasks you will need to see a computer screen. The head mask will have a mirror on it so you can see a computer screen behind you. There will be periods of time when the scanner is on and taking pictures of your brain and when the scanner is off. We will be able to talk to you in between scans (which last from 5-8 minutes) to check in on how you're doing.

Would you be comfortable in this kind of environment?

Notes, answer questions: ____

Now I'll tell you more about the rest of the set up in the scanner. After you lie down on the scanner platform, the scientist will move a structure over your head that will have a mirror attached so you can see the computer screen. They will put pads

around your head after you put in the ear plugs to help make your head comfortable and be stabilized.

Also, to measure heart and breathing rate, you will be wearing something on your finger as well as a belt around your waist. For some of the tasks, you will be asked to make responses by pushing a button. We will give you a button box with 4 buttons on it, and this will be placed under your right hand.

Do you think you can lay inside a tube for up to 2 hours with the set up I just explained? (mirror overhead, button box, finger and diaphragm attachments).

___ Yes ____ No (Stop) Notes____

Have you ever experienced claustrophobia or feeling anxious in small spaces? (even 1 time before is of concern)

Notes, answer questions: ____

- If concerned about claustrophobia, there is an option to schedule a 30-min appointment at the MRI scanner to have them test it out. Can continue with the rest of the screening if person is willing, and then schedule short appointment.

Do you have any other questions about anything I just explained?

Notes____

Assess Comfort and Noise

We will get you set up on the scanner bed first and make sure you are comfortable. You will lie down on a platform, and then will be moved into the middle of the scanner. You will be covered by a blanket, and we will give you cushions and bolsters for your back, neck, legs, and head. It's important for you to let us know what feels the best for you. When the scanner is turned on, it can make very loud noises. We will give you ear plugs to protect your ears. We will also give you a squeeze ball that you press at any point if you are uncomfortable or want to stop the scan for any reason.

Although we do our best to make the set up comfortable, you may experience discomfort over the course of the scan from the pressure of the pads or the loud noises you hear. Do you think you would be able to tolerate some discomfort if it happens?

___ Yes ____ No (Stop) Notes____

Will you be able to tolerate some loud sounds that may cause vibrations, be high-pitched, or be jarring? (Make sure no PTSD-like symptoms involving loud sounds)

___ Yes ____ No (Stop) Notes____

Assess Movement
It is very important for you to remain as still as you can when the scanner is on so we can get as clear pictures as possible. You can move if needed in between the scans for comfort and make slight adjustments (like moving your hand or toes). But in general, you need to remain very still.

Do you have any conditions that could impact your ability to lie still for 2 hours? For example, conditions that cause involuntary movements (like tremors), or conditions that prevent smooth breathing (frequent asthma attacks, coughing)

___ Yes ____ No (Stop) Notes____
If well-controlled, fine

Assess safety in the scanner
The MRI is in general safe, except in cases if there is magnetic metal in your body or on your body that cannot be removed. I will ask you more questions to make sure it is safe for you to be in the MRI scanner. (Administer MRI screening form, see attached. First, ask following questions)

a. Do you have any metal implants or devices in your body? Yes/No, Describe:

b. Do you have claustrophobia (fear of enclosed spaces)? Even once before? Yes/No/Describe

d. Do you have corrected vision? Yes/No
(if yes, clarify whether corrected vision is within +/- 8)

e. Do you wear glasses or contacts? Yes/no

If glasses, see if they have contacts. If they don't, they will wear goggles with lenses in the scanner – assess for comfort.

Can you read a computer screen without your glasses?
Yes/no

If yes, may not need to wear goggles in scanner.

Is there anything else we should be aware of regarding your mobility and safety in the MRI scanner? Do you have any other limitations that would make it difficult for you to lay in the scanner or complete the visual tasks?

___ Yes ____ No Notes_______________________________________

Assess ability to pay attention
During some of the scans, you can rest, and during other scans, we'll ask you to perform tasks. We will give you instructions for the tasks on the scan day before you go into the scanner. The tasks will involve paying attention to body sensations, thinking about what is going on in your day, a memory task with letters, and making some responses by pressing a button. Before each scan, we will let you know if you can rest or if there's a task as well as the length of the scan. Do you have any questions about that?

Notes____

Now I will ask you some more questions about your health to ensure you can participate in the MRI tasks, which requires lying still and paying attention to your experiences.

Assess for **<u>medical conditions</u>** that would prevent lying still, difficulty breathing or paying attention:

1. Do you have any current medical conditions? (for example, epilepsy, multiple sclerosis, cardiovascular disease, cancer, asthma, bronchitis). Ask further questions if any conditions may affect lying still or breathing
   Yes/No, Describe:

   Assess whether they will need assistance getting onto the MRI scanner bed

2. Do you have any medical condition(s) which impact your breathing (shortness of breath, labored breathing)? Examples include asthma, emphysema, chronic bronchial or pulmonary conditions and any other conditions that may impact your breathing.
   Yes/No, Describe:

3. Have you had an upper or lower respiratory tract infection in the past 5 weeks?
   Yes/No, Describe:

If Yes, have them wait before entering the study

4. Have you been diagnosed with sleep apnea?
   Yes/No, Describe:

   Check whether they could fall asleep in the scanner

5. Do you experience chronic pain?
   Yes/No, Describe:
   a. If yes, assess whether they would experience in the scanner. Assess current level of pain from 0 (no pain) to 10 (worst pain)

   Are you able to complete daily tasks?

   Have you noticed any changes in your ability to pay attention due to your pain?

   How do you feel your chronic pain affects your life on a daily basis?

6. Are you currently taking any medications?

   Type: _____________________ Dose: _______________________
   Type: _____________________ Dose: _______________________
   Type: _____________________ Dose: _______________________

Do any of these medications impact your attention when you take them, like make you drowsy? If yes:

Because ________ medication can impact your attention, would you be able to refrain from taking it for 24 hours before the experiment? Yes ____ No _____ (not included)

If taking medication for ADHD, should keep taking prescribed dose.

7. Are you currently experiencing any mental health concerns? (e.g., anxiety, depression, panic attacks, post-traumatic stress disorder, substance use disorder, attention deficit and hyperactivity disorder, bipolar disorder, schizophrenia, psychosis)
   Yes/No/Not Sure, Describe:

If currently experiencing mental health concerns:

Are you able to complete daily tasks?

Have you noticed any changes in your ability to pay attention?

How do you feel your mental health condition affects your life on a daily basis?

- If attention is affected, suggest that they participate at a later time.
- If report symptoms of schizophrenia and bipolar disorder, say: Unfortunately at this time, we are not including people who have experienced symptoms of schizophrenia or bipolar disorder because research shows they have differences in attention. This MRI task is currently under development, and we hope to be more inclusive of people in the future.

8. If not current, have you ever been treated for and/or experienced any mental health concerns?

   Yes/No/Not Sure, Describe:

- If report previous symptoms of schizophrenia and bipolar disorder, say: Unfortunately, at this time, we are not including people who have experienced symptoms of schizophrenia or bipolar disorder because research shows they have differences in attention. This MRI task is currently under development, and we hope to be more inclusive of people in the future.

9. In the past year, have you taken any psychiatric medications (medications to help with stress, sleep, anxiety, or depression) such as valium, diazepam, Ativan, clonazepam, Prozac, Lexapro, Zoloft, lithium, Ritalin, Adderall? Y/N

   If yes, ask the following:

   Have you made any changes to your psychiatric medications (type or dose) in the past month? Y/N, if Y, see if they can participate later after a month

If taking benzodiazepines, ask,

Are you able and willing to abstain from benzodiazepine use for 24 hours before the MRI?

Y/N/not sure If No, not included

10. Do you drink alcohol? Yes/Sometimes/No

If yes,

Are you able and willing to abstain from alcohol 24hrs before your mri?

Y/N; stop if N

11. Do you smoke tobacco? Yes/Sometimes/No

If yes,

How often do you smoke tobacco?

Do you experience frequent coughing and/or trouble breathing with or without your smoking?

If yes, assess how often it occurs. Discuss with person whether they think they would cough during the 2-hour MRI scan. If yes, not included in study.

12. Do you smoke marijuana or use edible marijuana?? Yes/Sometimes/No

If yes,

Are you able and willing to abstain from smoking marijuana and using edibles 24 hours before your MRI?

Y/N; stop if N

13. Have you used any other major recreational drugs, such as heroin, methamphetamine or cocaine in the past year?

Y/N

If yes, are you willing and able to refrain from taking major recreational drugs for a week before your MRI?

Y/N; stop if N

If yes, would it be medically safe for you to refrain for a week before the scan? Would you experience symptoms if you refrain? (Do not include if would experience difficult withdrawal symptoms) Consult with Dr. Weng if unsure

14. Is there anything else going on in your life that may impact your ability to lie still, or pay attention in the experiment?

(Clarify type of condition, length of illness, if interferes with daily functioning, if impacts feelings in the body, check with Dr. Weng and/or Dr. Hecht if participant is eligible for the study)

Pregnancy test for people who could be pregnant

For safety purposes, if you are not sure if you are pregnant, you will complete a pregnancy test to make sure you are not pregnant.

Are you still interested in participating in the MRI Scan?

If eligible, assess amount and type of meditation practice:

Now, I will ask you more specific questions about your meditation practice. First, I will ask about your personal practice, and then I will ask about retreat practice.

- Do you practice meditation from a particular tradition (e.g., Vipassana, Zen)?

  Y/N; if yes, list:

- Do you practice with a particular meditation group or sangha in addition to EBMC?

  List:

- What types of meditation do you practice? (General names and brief descriptions) Breath/Body?

- ○ ____ ☐
- ○ ____ ☐
- ○ ____ ☐
- ○ ____ ☐
- ○ ____ ☐

- Do any of these practices incorporate attention to the breath and/or body? If yes, which ones? (Check above, ask after each one is listed)

- Do you practice any of the following body-based meditation practices (ask if not already listed)?

  - Body Scan ☐
  - Visceral Sensations (emotions) ☐
  - Walking Meditation ☐
  - Any others (do not include yoga here) ☐

- How many years have you been engaged in a consistent meditation practice?
  - Most recent time period: Past _____ years
  - Previous time periods if there was a break in practice:

____

In the past _____ years of consistent practice, how much do you practice in a typical week? (If not specific): Can you estimate how many minutes/week that is?	__________ minutes
How much of that time is with a meditation class or group?	________ min
How much of that time is spent in personal practice?	________ min
Now I'm going to ask you what percentage of your practice focuses on your breath, what percentage focuses on your body, and what percentage focuses on something other than breath or body. The 3 different percentages should add up to 100.	
In a typical week, how much of your practice involves attention to breath sensations as your main focus?	____________ min<br><br>Or<br>______% of practice time
In a typical week, how much of your practice involves attention	____________ min

to body sensations as your main focus? (e.g., body scan, visceral sensations, walking)	Or<br>_______% of practice time
In a typical week, how much of your practice involves practices other than attention to breath sensations or body sensations as your main focus?	_____________ min<br><br>Or<br>_______% of practice time

- In previous years of practice (if there was a break), how much did you practice in a typical week?

_______________years	___________ min/week
How much of that time was with a meditation class or group?	________ min
How much of that time was spent in personal practice?	________ min
In a typical week, how much of your practice involved attention to<br>breath sensations?	_____________ min<br><br>Or<br>_______% of practice time
In a typical week, how much of your practice involved attention to body sensations? (e.g., body scan, visceral sensations, walking)	_____________ min<br><br>Or<br>_______% of practice time
In a typical week, how much of your practice involved practices other than attention to breath sensations or body sensations?	_____________ min<br><br>Or<br>_______% of practice time

- In previous years of practice (if there was a break), how much did you practice in a typical week?

_______________years	___________ min/week
How much of that time was with a meditation class or group?	________ min
How much of that time was spent in personal practice?	________ min
In a typical week, how much of your practice involved attention to<br>breath sensations?	_____________ min<br><br>Or<br>_______% of practice time
In a typical week, how much of your practice involved attention to body sensations? (e.g., body scan, visceral sensations, walking)	_____________ min<br><br>Or<br>_______% of practice time
In a typical week, how much of your practice involved practices	_____________ min<br><br>Or

other than attention to breath sensations or body sensations?	_______% of practice time

- Calculate total hours (RA does this)
  - Total hours of
    - All practices: ___________
    - Breath meditation: ___________
    - Body meditation: _____________
    - Other meditation: _____________

We have some more questions about your previous extended practice, which can include monastic practice, retreat practice, or taking a longer course at EBMC. I will send these questions to you as an online survey after we complete this call. We understand it may be difficult to remember exact times you have engaged in extended practice, so please give us your best estimate.

Monastic practice:

Did you ever have a monastic practice?	Yes No
If yes, for how many months or years did you have a monastic practice?	_____________ months/years
How much meditation practice did you engage in per day during this time?	______________ hours (try to only count formal practice)
How many of those hours included attention to breath sensations?	_____________ hours
How many of those hours included attention to body sensations?	_____________ hours

EBMC course practice (or other forms of extended practice):

In the past 5 years, did you take any courses at EBMC or another center? (estimate is fine)	Y/ N<br><br>Name/location of course:<br><br>Course Length: ______ hours/wk or mo<br><br>_______ years
How much meditation practice did you engage in on average?	______________ min/week (try to only count formal practice)
How many of those hours included attention to breath sensations?	_____________ min/week

How many of those hours included attention to body sensations?	____________ min/week

In the previous 5 years before that, did you take any other courses or had extended periods of practice?	Y/ N<br><br>Name/location of course:<br><br><br>Course Length: ______ hours/wk or mo<br><br>_______ years
How much meditation practice did you engage in per day on average?	_____________ min/week (try to only count formal practice)
How many of those hours included attention to breath sensations?	____________ min/week
How many of those hours included attention to body sensations?	____________ min/week

Keep going back 5 years as much as needed

________ days ________ hrs/day _______ hrs breath ___hrs body

________ days ________ hrs/day _______ hrs breath ___hrs body

Do you practice any other mind-body practices such as?

Yoga	_________ min / week for _________ years
Tai Chi	_________ min / week for _________ years
Other? _____________________________	_________ min / week for _________ years
Other? _____________________________	_________ min / week for _________ years

If any of these questions were answered in a way that prompted a "stop" command, the patient is not eligible for the study.

The interviewer would then say: "Thank you for the information you've just provided. Unfortunately, we cannot include you in our study because of MRI safety/ability to pay attention/etc. This study is under development, and we will work to make it more inclusive in the future. We thank you very much for your willingness to help in our research and we do wish you all the best. Thanks again and goodbye."

And this would end the screening interview.

Date: ____ / ____ / ____ Interviewer: ______________

If the criteria are met, the patient is eligible for the study.

The interviewer would then say: Thank you for the information. You are eligible for the study. I'd like to give you more details about the study, give you information about filling out questionnaires online, and set up a time you can visit the lab.

Can I record your full name at this time?

Full Name ________________________________

What is your email address?__________________

What is a good phone number to reach you?________________

Just in case we cannot reach you on this number, could you please give me an alternate number I can call you at?______________________________

[If not sure if eligible, tell participant we will check eligibility with study team and call them back.]

For further participation in the study, we require your verbal consent to participate. We will email or mail you a copy of the full consent form. We will then send you the retreat survey and the online questionnaires, and when you come into the lab we will go over the entire consent form with you before participating in the rest of the study.

Do you verbally consent for participation in this study?

________ Yes Verbal Consent ___________ No Verbal Consent

Online Questionnaires

We will email you a link to the online questionnaires for the study 1 week before your appointment (that we will schedule before the end of this call). We will include your participant number in the e-mail. Please only identify yourself in the surveys as your participant number. This will help keep your information confidential. Once you complete the surveys, you will be able to attend the scanning visit. The visit will take 3-3.5 hours. Generally, what is your schedule like?

Scheduling (Call participant time)

___ Get available NIC scanner times

___ Work out scanner time: Date: ____________ Time: ________________

____ They should arrive 1 hour before the start of the scan time

Instructions about Clothing

It is important to be comfortable in the scanner and to also be free from any metal. We suggest that you wear comfortable clothing such as sweat pants, yoga pants, t-shirts, and (for women) sports bras. You should avoid clothes that have metal such

as (for women) underwire bras, zippers, and metal buttons. We will ask you to remove anything with metal before the scan.

Travel

- Give options and assess how they will be getting to Mission Bay
- Give travel information for people with disabilities (free shuttle from BART 16th St to Mission Bay, parking for people with disabilities)
- All information included in follow up e-mail

"Again, thank you for your participation. Do you have any last questions for me?

Please feel free to call me at 415 514 8445 if you have any questions about the study at any time."

If none, thank participant and end call.

Checklist if eligible:

_____ Assign participant ID
_____ Send participant retreat survey
_____ Send participant online questionnaire link (1 week before scan date) and instructions, copy of consent form (electronic unless requested paper copies), directions to NIC, study reminders
_____ Get available scanner times. When participant finishes questionnaires, send detailed information about scan.
