# Supplementary material for: Toward a Compassionate Intersectional Neuroscience: Increasing Diversity and Equity in Contemplative Neuroscience
Source: Front Psychol. 2020 Nov 19;11:573134. doi: 10.3389/fpsyg.2020.573134 (PMC7711109; doi:10.3389/fpsyg.2020.573134)
Supplement: Supplementary file 3 [file Data_Sheet_3.PDF]

Demographics questionnaire

This demographics survey will be administered after the person has been determined to be eligible for the study. It will either be given over the phone or through an online survey, depending on the feedback we get from the East Bay Meditation Center community.

Instructions:
Thank you for participating in the EMBODY study with EBMC. For this survey, we will ask you to self-identify however you want without having to see lists of categories. Our goal is to use this information to increase awareness of the diversity of people who practice meditation by reporting this information in scientific articles and presentations. Because scientific papers tend to list identities in general categories, we may ask you some follow up questions to choose more general categories for scientific reporting purposes only. We will also ask about your education and financial situation.

All information we gather will be used in such a way as to protect your privacy and personal information. Please only share what you feel comfortable with, and you are free to decline answering any of the questions. We appreciate your help in our goals to increase diverse representation in neuroscience of meditation studies. If you have any suggestions about how to make this survey more inclusive and culturally sensitive, please let us know in person or by e-mailing embodystudy@ucsf.edu.

Demographic information

1. Participant ID: _______
2. What is your age? ______ years (dropdown: 25-65)
3. How do you self-identify your gender? [open box] and Do not wish to identify
    - Follow-up question for scientific reporting: *Some reviewers of this study may ask for the results to be presented with general categories, so we would like you to choose a broader category you may identify with. Recognizing that these categories may not fully represent your identity, with which general category do you most identify with?* [dropdown menu]
        - 1) cisgender male (gender assigned male at birth and identifies as male)
        - 2) cisgender female (gender assigned female at birth and identifies as female)
        - 3) another identity such as transgender, intersex, and/or non-binary genders
        - 4) Do not wish to specify
4. What pronouns do you use?
    - She/Her/Hers
    - He/Him/His
    - They/Them/Theirs
    - Other pronouns: __________________
    - Do not wish to specify

5. With which race or races do you identify? [open box]
    - Do you identify as multiracial?
        - Yes
        - No
        - If yes, please specify if you wish: [open box]

- For the purpose of scientific reporting, with which race(s) do you identify (please indicate all that apply)?
    - **American Indian or Alaska Native.** A person having origins in any of the original peoples of North and South America (including Central America), and who maintains tribal affiliation or community attachment.
    - **Asian.** A person having origins in any of the original peoples of the Far East, Southeast Asia, or the Indian subcontinent including, for example, Cambodia, China, India, Japan, Korea, Malaysia, Pakistan, the Philippine Islands, Thailand, and Vietnam.
    - **Black or African American.** A person having origins in any of the black racial groups of Africa.
    - **Native Hawaiian or Other Pacific Islander.** A person having origins in any of the original peoples of Hawaii, Guam, Samoa, or other Pacific Islands.
    - **White.** A person having origins in any of the original peoples of Europe, the Middle East, or North Africa.
    - **Multi-racial**: [open box]
    - **None of the above**
    - **Do not wish to specify**

6. With which ethnic identity or identities do you identify? Ethnicity refers to people who identify with each other based on similarities such as common ancestral, language, social, cultural, or national experiences. These can include shared cultural heritage, ancestry, origin myth, history, homeland, language or dialect, symbolic systems such as religion, mythology and ritual, cuisine, dressing style, art, and physical appearance. [open box]
    - Do you identify as multiethnic (of more than one ethnicity and/or heritage)?
        - Yes
            - Specify if you wish:
        - No
    - For purposes of scientific reporting, do you identify as Hispanic, Latinx, or of Spanish origin? (**Hispanic or Latinx or Spanish origin -** A person of Cuban, Mexican, Puerto Rican, Cuban, South or Central American, or other Spanish culture or origin, regardless of race)
        - Yes
        - No
        - Do not wish to report

7. How do you self-identify your sexual orientation? [open box] or Do not wish to identify
    - <u>**Follow-up question for scientific reporting</u>: *Some reviewers of this study may ask for the results to be presented with general categories. Recognizing that these categories may not fully represent your identity, with which general category do you most identify?* [dropdown menu]
        - lesbian/gay/homosexual
        - bisexual/pansexual
        - straight/heterosexual
        - asexual
        - Do not wish to specify

8. What is your religious or spiritual identity? [open box] or Do not wish to identify
9. Do you have any conditions that impact your learning, working or living activities

(such as physical disability, vision or hearing impairment, mobility condition, mental health condition, and/or learning disability)?

- Yes, please specify if you wish:
- No
- Do not wish to specify

10. How would you characterize your political views?

- Far left
- Liberal
- Moderate or middle of the road
- Conservative
- Far Right
- Undecided
- Other (please specify) ______________________________
- Do not wish to specify

11. What is the language(s) spoken in your home? List all that apply. [open box]

12. Are/were you a member of the U.S. armed forces?
    - I have not been in the military
    - Active military
    - Reservist
    - ROTC
    - Veteran
    - Do not wish to specify

13. Are there any other aspects of how you identify that you would like us to know about to report in scientific articles? [open box]

Socioeconomic information

14. What is your highest completed level of education?
    - No high school
    - Some high school
    - Completed high school/GED
    - Some college
    - Business/Technical certificate/degree
    - Associate's degree
    - Bachelor's degree
    - Some graduate work
    - Master's degree
    - Doctoral degree (Ph.D., Ed.D.)
    - Professional degree (e.g., MD, JD, MBA)
    - Do not wish to specify
15. What is your current work situation? (check all that apply)
    - Working for pay
    - Parental or sick leave
    - Retired
    - Unemployed and looking for work
    - Not working due to disability

- o Household manager
- o Student
- o Other (Specify) ______________________________
- o Do not wish to specify

16. If you are working for pay (part-time or full-time),
    - o On average, how many hours per week do you work in your main job? ___Hours
    - o If you have additional jobs, how many hours per week total do you work in those jobs? ___Hours
    - o What is your occupation in your main job? (specify)______________________________
    - o Do not wish to specify

17. If you are not now working for pay, have you ever had a regular job for pay?
    - o Yes
        - If yes, in what month and year did you stop working at your last regular job? _______
        - If yes, what was your occupation on that job? (Please be specific) ____
    - o No
    - o Do not wish to specify
18. Which of the sources listed below contributed to your household income over the past year? (mark all that apply)
    - o ___ Wages and salaries
    - o ___ Worker's compensation
    - o ___ Unemployment insurance
    - o ___ Government assistance (SSI, SSDI, TANF, WIC, Food Stamps, Other ___________)
    - o ___ Foster care payments
    - o ___ Child support or alimony
    - o ___ Dividends and interest on bonds, deposits, and savings certificates
    - o ___ Other: ______________________
    - o Do not wish to specify
19. What is your best estimate of your yearly household income, before taxes? Please include income from all family members living in your household and from all sources such as wages, salaries, commissions, pensions, family allowances, government assistance, child support, and so forth.
    - Below $10,000
    - $10,000-$19,999
    - $20,000-$29,999
    - $30,000 - $39,999
    - $40,000 - $49,999
    - $50,000 - $59,999

- $60,000- $69,999
- $70,000- $79,999
- $80,000 - $89,999
- $90,000- $99,999
- $100,000 - $124,999
- $125,000 - $149,999
- $150,000 - $199,999
- $200,000 - $249,999
- $250,000 - $299,999
- $300,000 - $399,999
- $400,000 - $499,999
- $500,000 or more

❍ Do not wish to specify

20. How many adults depend on this income?____________________________
21. How many children depend on this income? ________________________
22. Does anyone else depend on this income? ________________________
23. Do you support any pets? ________________________
24. Do you have health insurance?
    - o Yes
    - o No
    - o Do not wish to specify
