# Supplementary material for: Toward a Compassionate Intersectional Neuroscience: Increasing Diversity and Equity in Contemplative Neuroscience
Source: Front Psychol. 2020 Nov 19;11:573134. doi: 10.3389/fpsyg.2020.573134 (PMC7711109; doi:10.3389/fpsyg.2020.573134)
Supplement: Supplementary file 4 [file Data_Sheet_4.PDF]

UNIVERSITY OF CALIFORNIA, SAN FRANCISCO
CONSENT TO PARTICIPATE IN A RESEARCH STUDY

Study Title: EMBODY Study-Evaluating Multivariate MRI Maps of Body Awareness: A pilot functional magnetic resonance imaging (fMRI) investigation

Additional consent regarding sharing of brain data and demographic information

Thank you for participating in the UCSF EMBODY Study. This is an additional consent form regarding the option to share data with the scientific community including brain data, demographic information, and questionnaire data. There are many benefits to sharing your data with public databases and investigators. This sharing provides scientists with a pool of data from which they can make further scientific discoveries. Data sharing also benefits the field by enabling scientists to include diverse demographics in their studies that they may not have access to geographically. Larger and more inclusive samples help make scientific findings more accurate and reliable, for example a study of 2000 people is more representative of the general population than a study of 200 people.

Below, we will give you the option to share the different kinds of data collected in the EMBODY study including brain data (original data and/or analyzed data), demographic data, and questionnaire data. If you consent to share, these data may be posted to an open-access database. The data will be removed of identifying information such as name, birth date, the skull shape from brain image, height, and weight. However, we cannot guarantee total anonymity, privacy, and confidentiality.

In addition, we cannot fully guarantee that your data will not be re-identified. Individuals run the risk of re-identification if they have unique traits. Sometimes these are not identified until the study has been conducted. Brain scans may reveal differences in brain structure or function that may affect your health. These scans are not collected for clinical purposes and cannot be used for diagnoses. Furthermore, our study team is not trained to identify clinical problems. However, if we see something that may be of concern that requires further follow up from a physician, please initial here if you would like to be notified. Initials: _________________

Once data is posted publicly, it may not be able to be removed. If data is released to public neuroimaging online repositories (such as neurovault.org), it will likely be disseminated among scientific communities, and the information will be available even if data are removed from the website. We cannot foresee how the data will be used in the future, what questions the data will be used to answer, and what interpretations future researchers will make from the data. For example, based on similar kinds of brain structural data collected from the EMBODY study, researchers have been able to identify individuals with conditions such as depression[1], Alzheimer's disease[2], at risk mental states for psychosis and first episode psychosis[3], mild cognitive impairment[4], Autism spectrum disorder[5], schizophrenia[6], progressive supranuclear palsy and idiopathic Parkinson's disease[7]. Researchers have also been able to identify conditions based on resting state data similar to the data collected in the EMBODY study, for example;

Autism spectrum disorder[8], schizophrenia[9], Alzheimer's disease and mild cognitive impairment[10], major depression[11], attention deficit hyperactivity disorder (ADHD)[12].

Brain data consists of thousands of data points that can be analyzed in many different ways. **Original brain data** consists of brain structure and every data point from functional brain scans, and this data is at higher risk for being used to re-identify people. This data contains the original information from brain structure and function, and can be analyzed in new ways to answer different questions from the original study. **Analyzed brain data** consists of numbers derived from the original brain data for the EMBODY study, such as individualized brain maps, estimated time spent paying attention to the breath, and average brain activity from certain regions. This consists of less data that could be used to re-identify people. This data is oftentimes stored as numbers in a spreadsheet and can also include distilled brain maps.

Check here if **you do not want to share any data**:

- □ I do not wish to share any data from the study

Please check the boxes next to the data **you would like to share**:

Brain data:

- □ Analyzed brain data - metrics computed from EMBODY study (average brain activity, time spent paying attention to the breath)
- □ Analyzed brain data – individualized brain maps (higher risk for re-identification)
- □ Original brain data - all of the data for brain structure and function where new analyses can be conducted (higher risk for re-identification)

Questionnaire data:

- □ Bodily and emotional awareness
- □ Mindfulness
- □ Mental Health
- □ Physical Health

Please check which demographic information you consent to share. It should be noted, the more information that is shared, the higher the possibility for re-identification. Gender identity, age, race, and ethnicity are standard data points and are recommended if you consent to sharing of demographic data.

- □ Gender identity (recommended)
- □ Age (recommended)
- □ Race (recommended)
- □ Ethnicity (recommended)
- □ Sexual orientation
- □ Disability status
- □ Religious/Spiritual identity
- □ Political orientation
- □ Languages spoken at home
- □ Highest completed level of education

□ Employment status
□ Annual income, pre-taxes

Please sign below to indicate your consent to sharing the indicated brain, questionnaire, and demographic data from this study.

______________ ____

Date Participant's Signature for Consent

______________ ____

Date Person Obtaining Consent

Studies Cited:

[1] Costafreda, Sergi G., Carlton Chu, John Ashburner, and Cynthia H. Y. Fu. "Prognostic and Diagnostic Potential of the Structural Neuroanatomy of Depression." *PLOS ONE* 4, no. 7 (July 27, 2009): e6353. https://doi.org/10.1371/journal.pone.0006353

[2] Arimura, Hidetaka, Takashi Yoshiura, Seiji Kumazawa, Kazuhiro Tanaka, Hiroshi Koga, Futoshi Mihara, Hiroshi Honda, Shuji Sakai, Fukai Toyofuku, and Yoshiharu Higashida. "Automated Method for Identification of Patients With Alzheimer's Disease Based on Three-Dimensional MR Images." *Academic Radiology* 15, no. 3 (March 1, 2008): 274–84. https://doi.org/10.1016/j.acra.2007.10.020.

[3] Borgwardt, Stefan, Nikolaos Koutsouleris, Jacqueline Aston, Erich Studerus, Renata Smieskova, Anita Riecher-Rössler, and Eva M. Meisenzahl. "Distinguishing Prodromal From First-Episode Psychosis Using Neuroanatomical Single-Subject Pattern Recognition." *Schizophrenia Bulletin* 39, no. 5 (September 1, 2013): 1105–14.

[4] Davatzikos, Christos, Yong Fan, Xiaoying Wu, Dinggang Shen, and Susan M. Resnick. "Detection of Prodromal Alzheimer's Disease via Pattern Classification of Magnetic Resonance Imaging." *Neurobiology of Aging* 29, no. 4 (April 1, 2008): 514–23. https://doi.org/10.1016/j.neurobiolaging.2006.11.010.

[5] Ecker, Christine, Vanessa Rocha-Rego, Patrick Johnston, Janaina Mourao-Miranda, Andre Marquand, Eileen M. Daly, Michael J. Brammer, Clodagh Murphy, and Declan G. Murphy. "Investigating the Predictive Value of Whole-Brain Structural MR Scans in Autism: A Pattern Classification Approach." *NeuroImage* 49, no. 1 (January 1, 2010): 44–56. https://doi.org/10.1016/j.neuroimage.2009.08.024.

[6] Fan, Y., D. Shen, R. C. Gur, R. E. Gur, and C. Davatzikos. "COMPARE: Classification of Morphological Patterns Using Adaptive Regional Elements." *IEEE Transactions on Medical Imaging* 26, no. 1 (January 2007): 93–105. https://doi.org/10.1109/TMI.2006.886812.

[7] Focke, Niels K., Gunther Helms, Sebstian Scheewe, Pia M. Pantel, Cornelius G. Bachmann, Peter Dechent, Jens Ebentheuer, Alexander Mohr, Walter Paulus, and Claudia Trenkwalder. "Individual Voxel-Based Subtype Prediction Can Differentiate Progressive Supranuclear Palsy from Idiopathic Parkinson Syndrome and Healthy Controls." *Human Brain Mapping* 32, no. 11 (November 1, 2011): 1905–15. https://doi.org/10.1002/hbm.21161.

[8] Anderson, Jeffrey S., Jared A. Nielsen, Alyson L. Froehlich, Molly B. DuBray, T. Jason Druzgal, Annahir N. Cariello, Jason R. Cooperrider, et al. "Functional Connectivity Magnetic Resonance Imaging Classification of Autism." Brain: A Journal of Neurology 134, no. Pt 12 (December 2011): 3742–54. https://doi.org/10.1093/brain/awr263.

[9] Bassett, Danielle S., Brent G. Nelson, Bryon A. Mueller, Jazmin Camchong, and Kelvin O. Lim. "Altered Resting State Complexity in Schizophrenia." *NeuroImage* 59, no. 3 (February 1, 2012): 2196–2207. https://doi.org/10.1016/j.neuroimage.2011.10.002.

[10] Chen, Gang, B. Douglas Ward, Chunming Xie, Wenjun Li, Zhilin Wu, Jennifer L. Jones, Malgorzata Franczak, Piero Antuono, and Shi-Jiang Li. "Classification of Alzheimer Disease, Mild Cognitive Impairment, and Normal Cognitive Status with Large-Scale Network Analysis Based on Resting-State Functional MR Imaging." *Radiology* 259, no. 1 (April 2011): 213–21. https://doi.org/10.1148/radiol.10100734.

[11] Craddock, R. Cameron, Paul E. Holtzheimer, Xiaoping P. Hu, and Helen S. Mayberg. "Disease State Prediction from Resting State Functional Connectivity." *Magnetic Resonance in Medicine* 62, no. 6 (December 1, 2009): 1619–28. https://doi.org/10.1002/mrm.22159.

[12] Zhu, Chao-Zhe, Yu-Feng Zang, Qing-Jiu Cao, Chao-Gan Yan, Yong He, Tian-Zi Jiang, Man Qiu Sui, and Yu-Feng Wang. "Fisher Discriminative Analysis of Resting-State Brain Function for Attention-Deficit/Hyperactivity Disorder." *NeuroImage* 40, no. 1 (March 1, 2008): 110–20. https://doi.org/10.1016/j.neuroimage.2007.11.029.
