# Supplementary material for: Toward a Compassionate Intersectional Neuroscience: Increasing Diversity and Equity in Contemplative Neuroscience
Source: Front Psychol. 2020 Nov 19;11:573134. doi: 10.3389/fpsyg.2020.573134 (PMC7711109; doi:10.3389/fpsyg.2020.573134)
Supplement: Supplementary file 5 [file Data_Sheet_5.pdf]

Supplemental Information

Toward a compassionate intersectional neuroscience:
Increasing diversity and equity in contemplative neuroscience

Helen Y. Weng, Mushim P. Ikeda, Jarrod Lewis-Peacock, Duana Fullwiley, Maria T. Chao, Vierka Goldman, Sasha Skinner, Larissa G. Duncan, Adam Gazzaley, Frederick M. Hecht

Table of Contents

Page

Supplemental Methods and Results

Review of demographics in fMRI studies of meditation 2
Example of practicing cultural humility 3

EMBODY Step 1 – Internal Attention (IA) Task

Bodily regions of breath focus 4
Influence of head motion 4
Influence of respiration 4
Classifier confusion matrix 5
Subjective ratings instructions and classifier accuracy 6

EMBODY Step 3 – Attention metrics from meditation period

Preliminary group-level analyses 6

Supplemental Figures

Figure S1. Race and ethnicity in fMRI studies of meditation conducted in the U.S. 8
Figure S2. Frequency count of positive and negative importance voxels 9
Figure S3. Preliminary group-level attention metrics from meditation period 10

Supplemental Tables

Table S1. Respiration rate in the IA task 11
Table S2. Internal Attention (IA) task classifier confusion matrix 11
Table S3. Individual-level classification accuracies from the IA task 12
Table S4. Preliminary group-level meditation period metrics 12

Supplemental References 13

Review of demographics in fMRI studies of meditation.

Rationale. To learn which groups may be under-represented within fMRI studies of meditation, we conducted a review of racial and ethnic demographics from the literature. We also reviewed age and gender demographics.

Method. We reviewed demographics of participants included in a meta-analysis of functional neuroimaging studies of meditation (Fox et al., 2016), including age, gender, race, and ethnicity. Demographics were recorded for the 25 higher-quality studies used in the Fox et al. (2016) meta-analysis, where the main criteria were (i) reported specific peak foci of activation in Talairach or Montreal Neurological Institute (MNI) space, (ii) included a reasonable sample size, (iv) involved actual meditation during the scanning session, (v) involved healthy, non-clinical populations, and (vi) were published in peer-reviewed scientific journals. One study was removed because it was a positron emission tomography (PET) study (Lou et al., 1999), where the other studies used fMRI. To avoid counting the same participants more than once, studies that repeated the sample of another study were removed (n=2; Lutz et al., 2008, 2009). In addition, we included additional studies of lovingkindness and compassion meditation (reviewed in Weng et al., 2017) that met all previous criteria except they also included task-based fMRI data (vs. meditation session only) (Desbordes et al., 2012; Klimecki et al., 2012, 2014; Mascaro et al., 2013; Garrison et al., 2014; Hutcherson et al., 2014; Engen and Singer, 2015). This demographics review was conducted in 2016 and does not represent an exhaustive list of fMRI studies of meditation.

Two research assistants recorded demographics for the studies chosen, and any inconsistencies found were reviewed and corrected by the P.I. (H.Y.W.). If available, the demographic data included were age (mean, range), gender, race, ethnicity, and the study location. Because demographics were not always listed for meditators and controls separately, we report demographics representing full study samples. Because race and ethnicity are particularly salient aspects of identity in the U.S., demographics were particularly examined for U.S. studies where race and ethnicity were reported. We computed the percentage of participants within each racial and ethnic category defined by the U.S. National Institutes of Health (https://grants.nih.gov/grants/guide/notice-files/not-od-15-089.html). Race and ethnicity were not always administered as separate demographics in each study, so the denominator used for calculating percentages slightly differed (Race N=220, Ethnicity N=230). Finally, these percentages were compared to 2010 U.S. Census data (https://www.census.gov/prod/cen2010/briefs/c2010br-02.pdf). We formally tested the difference in racial and ethnic percentages between fMRI studies and the U.S. Census by using Chi-square tests in SPSS v. 24 (comparing frequency counts in fMRI vs. U.S. Census). Given that the Chi-square test requires a 2 x 2 cross tabulation, we compared frequency counts between samples of the following comparisons: White vs. Black or African American, White vs. Asian or Asian American, White vs. Other / Two or More Races, Hispanic or Latino vs. Non-Hispanic or Latino.

Results. Out of the 29 fMRI studies reviewed, 12 were conducted in the U.S., and 17 internationally, and 58.3% (7/12) of studies reported race and ethnicity data, representing 230 participants. Because of our particular focus on race and ethnicity in the U.S., we focused the analysis on these 230 participants (race was only in 17.6% or 3/17 of the international studies, and minority groups may differ by country and region). Within these participants, 84.09% (n=185) were White or Caucasian, 3.64% were Black or African American (n=8), 9.55% were Asian or Asian American (n=21), and 0.91% reported Other race (n=2; **Fig. S1**). No participants

identified as American Indian or Alaska Native, or Native Hawaiian and Other Pacific Islander. 6.07% of participants identified as Hispanic or Latino (n=14), and 83.7% did not (n=216). Using chi-square tests comparing the 2010 U.S. Census and fMRI studies of meditation, we found an under-representation of Black or African American vs. White Americans (Pearson chi-square=17.43, p=0.00003), an under-representation of Other races vs. White Americans (Pearson chi-square=19.22, p=0.0001), greater representation of Asian or Asian Americans vs. White Americans (Pearson chi-square=5.59, p=0.018), and an under-representation of Hispanic or Latino vs. Non-Hispanic or Latino (chi-square=17.42, p=0.00003) (**Fig. S1**). As no participants in fMRI studies were American Indian or Alaska Native, we did not formally test these groups.

The mean age across all studies (Participant N=737) was 38.92, with ages ranging from 18-69. These data were consistent across U.S. (Participant n=316; mean=39.3, range=18-69) and International (Participant n=421, mean=38.7, range=18-68) studies. Across all studies, gender appeared relatively equivalent (Male=49.3%, Female=50.7%). However, looking at U.S. studies separately from International studies showed differential rates of gender inclusion (U.S.: Male=54.4%, Female=45.6%; International: Male=45.4%, Female=54.6%). Further, no study reported including transgender or other gender identities.

Discussion. Comparing racial and ethnic demographics between fMRI studies of meditation and the 2010 U.S. Census, we found a significant under-representation of Black or African Americans, those identifying as an Other race, and Hispanic or Latino people. Further, no people identifying as American Indian or Alaska Native, Native Hawaiian and Other Pacific Islander, or Multiracial were included. We found a significant over-representation of Asian or Asian American people in fMRI studies, although this is to be expected as meditation comes from Asian cultures, and monks from Asian countries were included in U.S. studies. Potential disparities of inclusion for Asian-Americans should be further investigated. In addition, 41.7% of U.S. studies did not report race and ethnicity data at all. We recommend all studies within the U.S. report race and ethnicity data, and other regions should consider which minority groups should be reported given their cultural context. Women and non-binary gender identities may be under-represented in U.S. studies. Researchers should examine their study procedures for recruitment and screening (and consider employing community engagement as described in this paper) to reduce these disparities and increase generalizability of results.

Example of practicing cultural humility.

Conducting community-engaged research is characterized by practicing cultural humility (Tervalon and Murray-García, 1998; Wallerstein and Duran, 2010). Below is an excerpt from an early e-mail communication at the beginning of developing a community partnership with the East Bay Meditation Center in Oakland, CA. The e-mail communicates that the researcher is aware of historical inequities between scientists and under-represented populations, and is open to any feedback about interactions that may be replicating these patterns. The researcher also expresses that she brings awareness to communication as a form contemplative practice, which indicates self-awareness around communication style and the ability to change as needed.

E-mail excerpt: "I also want to express that I am aware that there is a history of the scientific and academic communities sometimes taking advantage of underrepresented communities, and I want to make sure these dynamics are not repeated with any interactions we have with EBMC. I am open to any dialogue regarding these dynamics if they appear to be at play, and I approach communication as a form of contemplative practice."

EMBODY Step 1: Internal Attention (IA) task.

Bodily regions of breath focus. Before the MRI scan, participants were instructed to notice where they felt the breath the most strongly in their body, and instructed to keep their attention in that bodily region for the IA task. Participants chose the nostrils (n=5), throat (n=2), chest (n=1), stomach (n=6), and diaphragm (n=1).

Influence of head motion. To investigate the potential influence of head motion (Churchill et al., 2012), we compared motion in each of 5 conditions in the IA task (Breath, Feet, MW, Self, and Sounds). Using the 1D files output by AFNI's 3dvolreg,we computed mean motion in each of 6 directions (roll, pitch, yaw, superior-inferior [dS], left-right [dL], posterior-anterior [dP]) as well as total mean motion across all 6 directions (7 metrics total). Difference in motion between all 5 conditions were tested using a one-way ANOVA for each direction and total motion. ANOVAs were also computed for the 3 main conditions of interest only (Breath, MW, Self). Head motion did not vary between the 5 conditions in any of 7 motion metrics (all one-way ANOVA $Fs_{4,74}<0.66$, $ps>0.62$), nor between the 3 conditions relevant for meditation (Breath, MW, Self; all one-way ANOVA $Fs_{2,44}<0.85$, $ps\geq0.43$).

The first EMBODY task study with 14 participants (Weng et al., 2020) showed that classifier accuracy was not substantially driven by motion, and all classifier accuracies remained robust for each condition after covarying head motion out of the BOLD signal using 3dDetrend. We therefore did not correct for head motion in the main analysis of this study. However, to mitigate the impact of individual-level head motion on identifying voxels in the importance map analyses, we conducted the importance map analysis on fMRI data with head motion covaried out (**Figs. 3** and **4** in main manuscript; **Fig. S2**).

Influence of respiration. Previous research demonstrates respiration rate may change depending on meditative state and practice experience (Farb et al., 2013; Wielgosz et al., 2016), which may subsequently influence blood-oxygen-level dependent (BOLD) fMRI activity. Before fully discussing the influence of respiration in this experiment, it is important first to clarify the conceptual and methodological differences between standard univariate approaches and our multivariate approach (MVPA) in analyzing fMRI data to study meditation. In the standard univariate approach, brain data from different cognitive states (e.g., meditation vs. rest) are compared and averaged for the purpose of *brain mapping*, or identifying brain regions associated with mental states at the group level. In this case, the location in the brain is the main outcome, and therefore it would be important to regress out activation due to non-neural sources such as respiration. However, in our multivariate approach with the EMBODY framework, we are instead using the brain data at the individual level to *distinguish and decode cognitive states* during meditation (Norman et al., 2006). The resulting *cognitive states are the main outcome of interest* rather than brain activity *per se*. Therefore, any source of information that contributes to differential brain patterns in the BOLD signal are useful (such as physiology), and should *not* be regressed out as they would remove important diagnostic signals.

We therefore frame our investigation of the influence of respiration as secondary analyses to understand whether 1) respiration rate changes due to IA task instruction (particularly for Breath, MW, and Self), and 2) accurate classification accuracy in the IA task is primarily driven by changes in respiration signal.

Methods. Respiration data were collected using a Siemens respiration belt at a sampling rate of 51 Hz. Full respiration datasets were available for only 6/15 participants due to technical

errors (such as faulty respiration belt signal or incomplete transfer of data). With this subsample, we examined the influence of internal attention on respiration rate (RR) at the trial level within each subject using in-house Python software. The raw respiration data were linearly detrended and processed with a low-pass filter (5th-order Butterworth filter) with a cut-off frequency of 0.5 Hz. Peaks and troughs were identified as local maxima and minima within the respiratory signal. Respiratory phase was modeled by coding peaks (1), troughs (-1), and neither peak nor trough (0). The number of breaths were counted per trial as trough-to-trough intervals, and converted to respiration rate (RR) in breaths per minute for the main conditions of interest: Breath, MW, and Self.

Results. Within each subject, mean RR between each pair of conditions was tested with paired *t*-tests using the first 12 trials in each condition (18 breath trials were administered at shorter durations, so only the first 12 trials were included). Because this analysis is exploratory to examine whether internal attention influences RR, we did not correct for multiple comparisons and report all tests for completeness and to encourage further research. Half of participants (3/6) showed slower RR for Breath vs. MW (t_{11}s<-2.46, *p*s<0.05), and 2/6 showed slower RR for Breath vs. MW near or at trend level (t_{11}s<-1.76, *p*s<0.11). 4/6 participants showed slower RR for Breath vs. Self (*t*s<-2.57, *p*s<0.05), and one participant showed this at trend-level ($p<0.10$). Only 1/6 participants showed a significant difference in RR between MW vs. Self ($t_{11}=-2.87$, $p<0.05$). Subject-level data are reported in **Table S1.** We also did exploratory *t*-tests using the mean RR of each condition in each subject (N=6), and found that the mean RR for Breath was significantly lower compared to MW ($t_5<-3.50$, $p=0.02$) and Self ($t_5<-3.68$, $p=0.01$).

Because differences in RR between conditions were found, we regressed the processed respiration waveform from the fMRI BOLD data using AFNI's 3dRetroIcor (before slice-time correction, as recommended by AFNI), and found that Step 1 Internal Attention task classifier accuracy remained similar to the primary analysis without respiration covaried out (mean difference = 0.24%, range of -5.09% to 6.71%). Individual-level classification accuracy was tested using Chi-Square tests, and yielded the same significance result in each condition in each subject as the primary analysis (5 subjects showed significant accuracy in all 3 conditions, 1 subject did not have accurate classification in MW). For this small sample, the results suggest that classifier accuracy is *not* primarily driven by variations in respiratory signals, and this is consistent with the findings in the first study (Weng et al., 2020; n=3 with respiration data). However, future studies should investigate these questions more rigorously, and investigate how decoded mental state transitions during meditation relate to temporal parameters of physiology.

Classifier confusion matrix.

The classifier confusion matrix consists of the number of classifier decisions for each category given the instructed condition in the IA task (**Table S2**), averaged across all 16 participants. 432 total decisions were made for each second of data in each category in the IA task (see Methods for full classification details using MVPA (Norman et al., 2006). In each condition, the greatest number of classifier decisions were made for the correct congruent category (**Table S2**), indicating that the classifier could accurately recognize the brain pattern for each condition. Breath brain patterns were most likely to be confused with Feet brain patterns, indicating confusion with attention to another area of the body. Mind Wandering (MW) brain patterns were most likely to be confused with listening to ambient MRI sounds (Sounds) brain patterns (suggesting mind wandering may be related to becoming distracted by sounds), whereas Self brain patterns were most likely to be confused with Feet brain patterns. Finally, the control

conditions of Feet and Sounds were most likely to be confused with each other. Mean classifier accuracies and SD of each condition are also listed in **Table S2** to aid future research (displayed in **Fig. 2b** in the main manuscript).

Subjective ratings instructions and classifier accuracy. Attention ratings were collected for the second half of trials (33/39 trials, excluding MW trials where no rating was administered). To estimate the correlation of classifier accuracy and ratings within each subject, a Pearson's correlation was computed between trial-level classifier accuracy and subjective ratings of attention. To test the strength of correlations across the group, each individual *r*-value was transformed using a Fisher *r*-to-*Z* transform, and the group mean *Z*-score was tested vs. 0 using a one-sample *t*-test. Because this task was designed to measure breath attention, we also examined accuracy-rating correlations specifically in Breath trials (n=9).

In this study, we did not see significant within-subjects correlations between subjective ratings of attention and classifier accuracy, for either all conditions (mean Z=0.04, p=0.41; MW trials not included because participants were explicitly told to stop paying attention) or breath trials only (mean Z=0.07, p=0.48). This is in contrast to the first study (Weng et al., 2020) where we saw a significant positive relationship between attention ratings and classifier accuracy for all conditions as well as breath trials only. This may be due to differences in instructions for the ratings task in the current study. In the first study, participants were instructed to make their best efforts in using all 4 ratings (1=less attention to 4=more attention) in the task. Many participants had difficulty in using all 4 ratings, particularly for experienced meditators who tended to indicate strong attention throughout the task. For the current study, we changed the instructions and de-emphasized the need to use all 4 ratings. We instructed participants to press "3" if their attention was relatively strong, press "2" if attention was less strong (emphasizing use of these 2 ratings), and then to only press "4" or "1" if attention was noticeably better or worse. Participants could complete the task more easily, but it likely decreased the variability in ratings and decreased the power to detect correlations. For example, many participants would choose only "3" or "4", particularly given they were experienced meditators. Further pilot testing should be done with ratings instructions and task design such as using a ratings dial.

EMBODY Step 3: Attention metrics from meditation period

Step 3: Quantify internal attention metrics during meditation. From the mental state classifications from Step 2, novel metrics of internal attention during meditation were computed for each participant. For each mental state, *percentage time engaged, number of events, mean duration of events*, and *variability* (SD) of event duration were computed. Data were preliminarily analyzed at the group level by testing for differences in metrics between conditions (Breath, MW, Self) with a one-way ANOVA. To test our main hypotheses that breath-focused meditation would result in differences between Breath vs. other mental states, significant results were followed up with planned pair-wise *t*-tests of Breath vs. MW and Breath vs. Self. Data were analyzed in SPSS (v. 24), figures were created with R, and brain maps were displayed using AFNI.

Preliminary group-level analyses of attention metrics during the meditation period. Although this study focuses on the capacity for the EMBODY task to capture individual-level brain patterns and metrics, attention profiles from the 10-min meditation period were preliminarily characterized at the group level to examine initial hypotheses about mental states during meditation. For breath-focused meditation, we hypothesized that participants would direct

their attention more to the breath than engaging in mind wandering or self-referential processing. Therefore, compared to the other mental states, participants should show greater: 1) percentage time attending to the breath, 2) number of breath mental events, 3) mean duration of attention to the breath, and 4) variance in duration on the breath (greater inter-trial variability due to longer durations). Data were preliminarily analyzed at the group level by testing for differences in metrics between conditions (Breath, MW, Self) with a one-way ANOVA. To test our main hypotheses that breath-focused meditation would result in differences between Breath vs. other mental states, significant results were followed up with planned pair-wise *t*-tests of Breath vs. MW and Breath vs. Self.

Attention metrics differed in the percentage time engaged in each mental state ($F_{2,42}$=4.46, p=0.018), but not the mean duration of mental events ($F_{2,42}$=1.41, p=0.25), or the mean duration or variance of event durations ($F\mathrm{s}_{2,42}$<1.41, *ps*>0.25; **Fig. S3**). Consistent with our hypotheses, we found that during meditation, participants spent more time paying attention to their breath compared to mind wandering (t_{14}=2.27, p=0.04) and self-referential processing at trend level (t_{14}=1.76, p<0.10). Planned contrasts of mean duration of events showed that the mean duration of breath events was greater than the mean duration of mind wandering (t_{14}=2.21, p=0.044) but not self-referential processing (p=0.94; **Fig. S3**). See **Table S4** for all group-level attention metric statistics.

Discussion. Participants showed greater percentage time attending to the breath vs. mind wandering, as well as greater mean duration on the breath vs. mind wandering. This replicates the finding in Weng et al. (2020) and supports the interpretation that participants are indeed able to engage longer on the breath during breath-focused meditation: when their attention is on the breath, it tends to stay there longer compared to mind wandering. This suggests that meditators were able to implement and maintain the meditative goal of focusing on the breath. However, this needs to be tested in larger samples. Further, participants may have been able to focus longer on the breath due to being a highly educated sample rather than due to meditation training *per se*, where they may have greater cognitive abilities in general. and potentially showing greater cognitive abilities overall. Future studies with larger samples can empirically test these points by including participants with a wider range of socioeconomic status, and employing longitudinal designs which can test the impact of meditation training.

Supplemental Figures

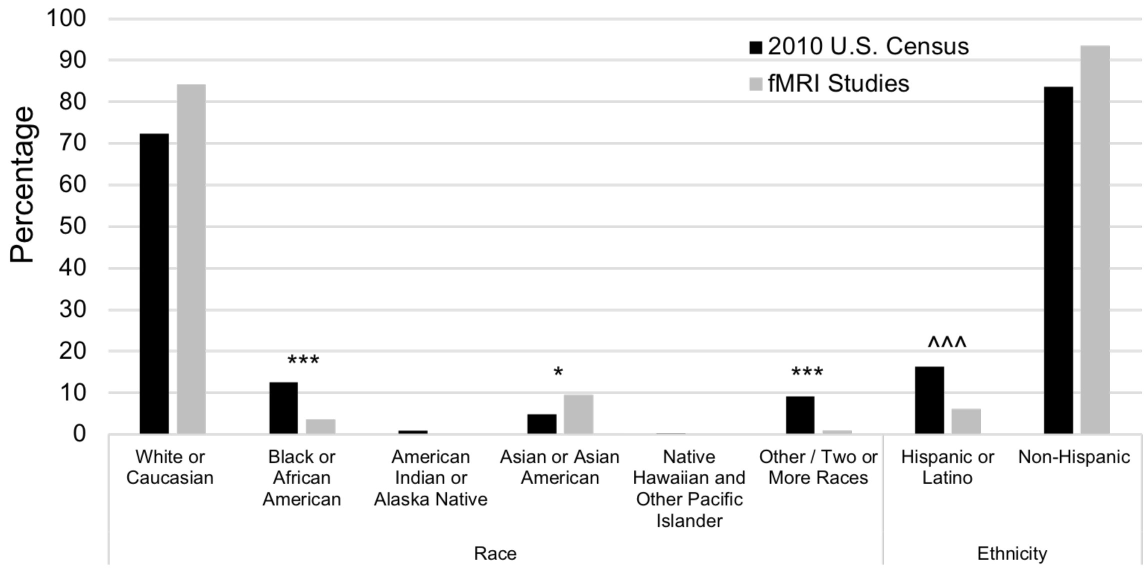

Figure S1. Race and ethnicity in U.S. fMRI studies of meditation vs. 2010 U.S. Census.
* Pearson chi-square=5.59, $p<0.05$ compared to White or Caucasian Americans
*** Pearson chi-square>17.43, $p<0.001$ compared to White or Caucasian Americans
^^^ Pearson chi-square=17.42, $p<0.001$ compared to Non-Hispanic or Latino

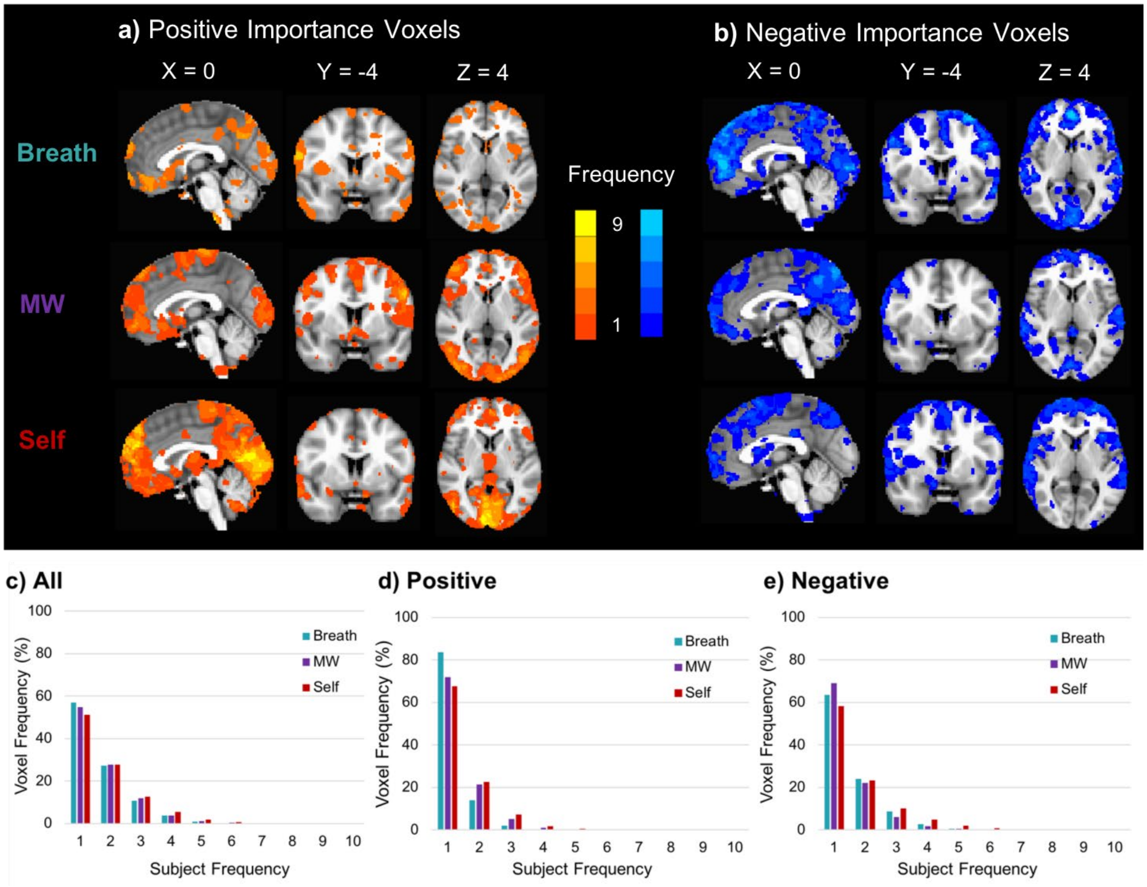

Figure S2. Frequency count of positive and negative importance voxels, identified using fMRI data with the influence of head motion removed. **Fig. 3b** from the main manuscript displays the frequency map of all importance voxels across 15 participants, regardless of the direction of average *z*-scored activation of the voxels. This figure displays the frequency maps specific for **a)** "positive importance" voxels, which have a positive weight and positive *z*-scored average activation value (indicating that it was more active on average, displayed in warm colors), and **b)** "negative importance" voxels, which had a negative weight and a negative *z*-score average activation value (indicating that it was less active on average, displayed in cool colors) (McDuff et al., 2009). The frequency indicates the number of subjects for which that voxel was important in distinguishing the mental state (Positive maximum for Breath = 5/15, MW = 9/15, Self = 6/15; Negative maximum for Breath = 6/15, MW = 8/15, Self = 8/15). Histograms are displayed for the percentage of voxels at each subject frequency (1-10) for **c)** all, **d)** positive, and **e)** negative importance voxels. Most importance voxels contained a subject frequency of 1 or 2.

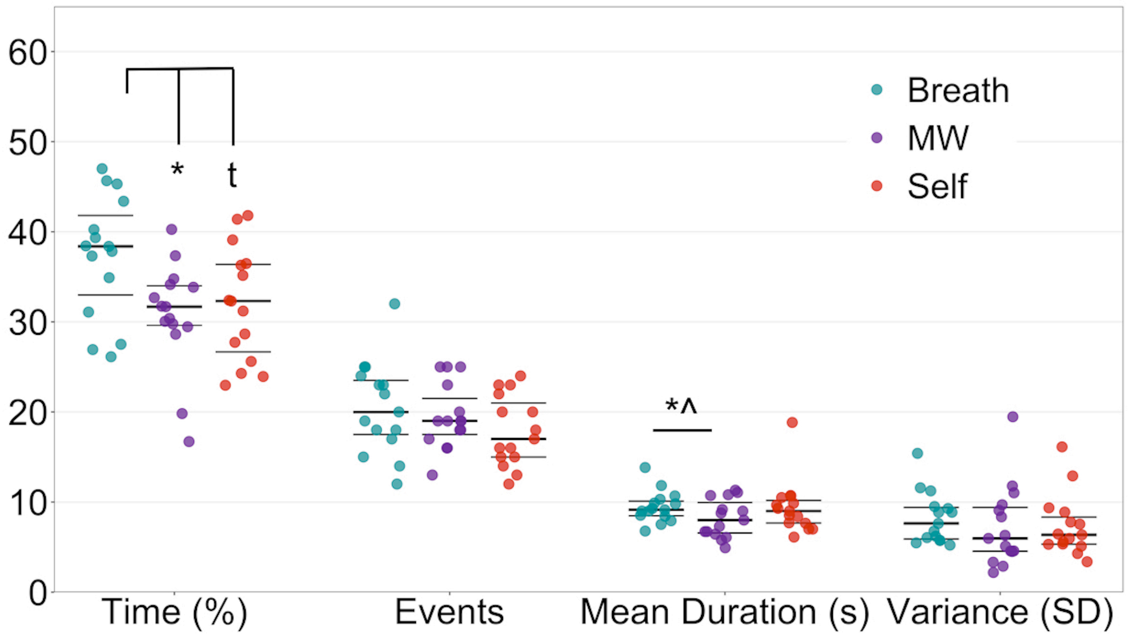

Figure S3. Preliminary group-level attention metrics from meditation period. Based on the estimate of mental states and event specification from Step 2, metrics of attention during breath meditation were quantified for each mental state and initially characterized at the group level (N=15): percentage time spent in each mental state (Breath, MW, or Self), the number of events, mean duration of events (s), and variability (standard deviation or SD) of duration of events. Overall, participants spent more time attending to the breath vs. mind wandering. Beeswarm plots present each data point, the median (bold black line), and ±25th percentile range (gray lines). See **Table S4** for full metric statistics.

* paired t_{14}=2.27, p=0.04, ^{t} paired t_{14}=1.76, p<0.10), after one-way ANOVA $F_{2,42}$=4.46, p=0.018

*^ Planned contrast: paired t_{14}=2.21, p=0.044

Supplementary Tables

	Respiration Rate			Paired t_{11}-tests		
	Breath	MW	Self	Br vs. MW	Br vs. Self	MW vs. Self
**Ppt 1**	8.7 (2.9)	11.6 (2.1)	10.5 (2.2)	-3.04*	-1.91^t	1.16
**Ppt 2**	14.8 (3.0)	21.7 (2.0)	20.5 (2.0)	-6.22***	-5.67***	1.43
**Ppt 3**	7.5 (2.9)	9.8 (3.2)	10.5 (3.4)	-2.47*	-2.67*	-0.96
**Ppt 4**	13.6 (1.3)	14.6 (2.0)	15.6 (1.7)	-1.51	-2.87*	-2.40*
**Ppt 5**	10.4 (3.3)	13.1 (3.7)	12.1 (4.4)	-1.77	-1.16	0.79
**Ppt 6**	13.9 (4.4)	17.2 (3.3)	17.8 (2.4)	-1.87^t	-2.57*	-0.81
				**Paired t_5-tests**		
**Group mean**	11.8 (2.9)	14.7 (4.3)	14.5 (4.1)	-3.50*	-3.68*	0.30

Table S1. Respiration rate in the IA task for 6 participants. For each participant, mean respiration rate (SD) for each condition relevant for meditation in the Internal Attention task (12 trials each), including paired $t_{1,11}$-test values for each condition pair. Group means and paired *t*-tests are also reported. These data are exploratory, and are not corrected for multiple comparisons. ^{t}<0.01, * p<0.05, ** p<0.01, *** p<0.001

		Decisions						
		Breath	MW	Self	Feet	Sounds	Accuracy	SD
**Condition**	Breath	**224.1**	44.2	50.1	*67.3*	46.3	51.9****	18.6
	MW	56.3	**211.3**	49.9	56.9	*57.6*	48.9***	22.6
	Self	52.6	41.9	**216.3**	*61.7*	59.5	50.1***	12.7
	Feet	70.9	58.0	60.5	**165.9**	*76.7*	38.4****	14.3
	Sounds	60.2	55.1	64.2	*90.1*	**162.5**	37.6****	11.8

Table S2. Internal Attention (IA) task classifier confusion matrix. For all 15 participants in the IA task from Step 1, the mean decisions made in each category given each instructed condition are reported. **Bolded** numbers indicate the correct classifier decisions made for each instructed condition. *Italicized* numbers indicate the condition for which each instruction category was most likely to be "confused" with. Accuracy indicates the mean classifier accuracy for each instructed condition, and SD indicates the standard deviation of classifier accuracy.
*** $p < 0.001$ vs. 20% (theoretical chance)
**** $p < 0.0001$ vs. 20% (theoretical chance)

		Internal Attention Neural Pattern		
		Breath	MW	Self
p value	< 0.001	15	11	15
	< 0.01	0	1	0
	< 0.05	0	0	0
	> 0.05	0	3	0

Table S3. Individual-level classification accuracies from the Internal Attention (IA) task. The table indicates the number of participants for which each neural pattern relevant for breath-focused meditation (from Step 1 of the IA task) was recognized above chance within the individual. Individual-level accuracy was determined using a Chi-square test comparing the frequency of correct vs. incorrect MVPA decisions for each mental state compared to chance distribution (87 correct vs. 345 incorrect decisions). Participant frequencies are displayed for varying p-values. In order to use the neural patterns for subsequent decoding the meditation period (Step 2), we required that each individual have at least 2/3 brain patterns significantly identified above chance ($p < 0.05$). 15/15 participants survived this criterion, who were all experienced meditators. Most of these individual-level brain patterns were distinguished at a high significance ($p < 0.001$).

	**EMBODY Meditation Period Metrics – Mean (SD)**				
	Percentage Time	Number of events	Mean duration	Variance (SD)	Total events
**All (N=14)**					57.8 (8.9)
Breath	37.3 (6.8)	20.5 (5.2)	9.5 (1.8)	8.2 (2.9)	
MW	30.8 (6.0)	19.5 (3.6)	8.2 (2.1)	7.3 (4.5)	
Self	32.0 (6.3)	17.9 (1.0)	9.4 (3.0)	7.4 (3.4)	

Table S4. Preliminary group-level meditation period metrics. Means and SDs for meditation period metrics for all participants with distinguishable brain patterns used to decode the meditation period (N = 15/15). Mean duration and variance of mean duration (SD) are reported in seconds.

Supplemental References

Churchill, N. W., Oder, A., Abdi, H., Tam, F., Lee, W., Thomas, C., et al. (2012). Optimizing preprocessing and analysis pipelines for single-subject fMRI. I. Standard temporal motion and physiological noise correction methods. *Hum. Brain Mapp.* 33, 609–627. doi:10.1002/hbm.21238.

Desbordes, G., Negi, L. T., Pace, T. W. W., Wallace, B. A., Raison, C. L., and Schwartz, E. L. (2012). Effects of mindful-attention and compassion meditation training on amygdala response to emotional stimuli in an ordinary, non-meditative state. *Front. Hum. Neurosci.* 6, 292. doi:10.3389/fnhum.2012.00292.

Engen, H. G., and Singer, T. (2015). Compassion-based Emotion Regulation Up-regulates Experienced Positive Affect and Associated Neural Networks. *Soc. Cogn. Affect. Neurosci.* 10, 1291–1301. doi:10.1093/scan/nsv008.

Farb, N. A. S., Segal, Z. V., and Anderson, A. K. (2013). Mindfulness meditation training alters cortical representations of interoceptive attention. *Soc. Cogn. Affect. Neurosci.* 8, 15–26. doi:10.1093/scan/nss066.

Fox, K. C. R., Dixon, M. L., Nijeboer, S., Girn, M., Floman, J. L., Lifshitz, M., et al. (2016). Functional neuroanatomy of meditation: A review and meta-analysis of 78 functional neuroimaging investigations. *Neurosci. Biobehav. Rev.* 65, 208–228. doi:10.1016/j.neubiorev.2016.03.021.

Garrison, K. A., Scheinost, D., Constable, R. T., and Brewer, J. A. (2014). BOLD signal and functional connectivity associated with loving kindness meditation. *Brain Behav.* 4, 337–347. doi:10.1002/brb3.219.

Hutcherson, C. A., Seppala, E. M., and Gross, J. J. (2014). The neural correlates of social connection. *Cogn. Affect. Behav. Neurosci.*, 1–14. doi:10.3758/s13415-014-0304-9.

Klimecki, O. M., Leiberg, S., Lamm, C., and Singer, T. (2012). Functional Neural Plasticity and Associated Changes in Positive Affect After Compassion Training. *Cereb. Cortex* 23, 1552–1561. doi:10.1093/cercor/bhs142.

Klimecki, O. M., Leiberg, S., Ricard, M., and Singer, T. (2014). Differential pattern of functional brain plasticity after compassion and empathy training. *Soc. Cogn. Affect. Neurosci.* 9, 873–879. doi:10.1093/scan/nst060.

Lou, H. C., Kjaer, T. W., Friberg, L., Wildschiodtz, G., Holm, S., and Nowak, M. (1999). A 15O-H2O PET study of meditation and the resting state of normal consciousness. *Hum. Brain Mapp.* 7, 98–105.

Lutz, A., Brefczynski-Lewis, J., Johnstone, T., and Davidson, R. J. (2008). Regulation of the neural circuitry of emotion by compassion meditation: effects of meditative expertise. *PloS One* 3, e1897. doi:10.1371/journal.pone.0001897.

Lutz, A., Greischar, L. L., Perlman, D. M., and Davidson, R. J. (2009). BOLD signal in insula is differentially related to cardiac function during compassion meditation in experts vs. novices. *NeuroImage* 47, 1038–1046. doi:10.1016/j.neuroimage.2009.04.081.

Mascaro, J. S., Rilling, J. K., Negi, L. T., and Raison, C. L. (2013). Compassion meditation enhances empathic accuracy and related neural activity. *Soc. Cogn. Affect. Neurosci.* 8, 48–55. doi:10.1093/scan/nss095.

McDuff, S. G. R., Frankel, H. C., and Norman, K. A. (2009). Multivoxel Pattern Analysis Reveals Increased Memory Targeting and Reduced Use of Retrieved Details during Single-Agenda Source Monitoring. *J. Neurosci.* 29, 508–516. doi:10.1523/JNEUROSCI.3587-08.2009.

Norman, K. A., Polyn, S. M., Detre, G. J., and Haxby, J. V. (2006). Beyond mind-reading: multi-voxel pattern analysis of fMRI data. *Trends Cogn. Sci.* 10, 424–430. doi:10.1016/j.tics.2006.07.005.

Tervalon, M., and Murray-García, J. (1998). Cultural Humility Versus Cultural Competence: A Critical Distinction in Defining Physician Training Outcomes in Multicultural Education. *J. Health Care Poor Underserved* 9, 117–125. doi:10.1353/hpu.2010.0233.

Wallerstein, N., and Duran, B. (2010). Community-Based Participatory Research Contributions to Intervention Research: The Intersection of Science and Practice to Improve Health Equity. *Am. J. Public Health* 100, S40–S46. doi:10.2105/AJPH.2009.184036.

Weng, H. Y., Lewis-Peacock, J. A., Hecht, F. M., Uncapher, M. R., Ziegler, D., Farb, N. A. S., et al. (2020). Focus on the breath: Brain decoding reveals internal states of attention during meditation. *Front. Hum. Neurosci.* 14. doi:10.3389/fnhum.2020.00336.

Weng, H. Y., Schuyler, B., and Davidson, R. J. (2017). The Impact of Compassion Meditation Training on the Brain and Prosocial Behavior. *Oxf. Handb. Compassion Sci.* doi:10.1093/oxfordhb/9780190464684.013.11.

Wielgosz, J., Schuyler, B. S., Lutz, A., and Davidson, R. J. (2016). Long-term mindfulness training is associated with reliable differences in resting respiration rate. *Sci. Rep.* 6. doi:10.1038/srep27533.
